# *Staphylococcus aureus* membrane vesicles contain immunostimulatory DNA, RNA and peptidoglycan that activate innate immune receptors and induce autophagy

**DOI:** 10.1002/jev2.12080

**Published:** 2021-04-01

**Authors:** Natalie J. Bitto, Lesley Cheng, Ella L. Johnston, Rishi Pathirana, Thanh Kha Phan, Ivan K. H. Poon, Neil M. O'Brien‐Simpson, Andrew F. Hill, Timothy P. Stinear, Maria Kaparakis‐Liaskos

**Affiliations:** ^1^ Department of Physiology Anatomy and Microbiology La Trobe University Melbourne Victoria 3086 Australia; ^2^ Research Centre for Extracellular Vesicles School of Molecular Sciences La Trobe University Melbourne Victoria 3086 Australia; ^3^ Department of Biochemistry and Genetics, La Trobe Institute for Molecular Science La Trobe University Melbourne Victoria 3086 Australia; ^4^ Centre for Oral Health Research Melbourne Dental School Bio21 Institute The University of Melbourne Parkville Victoria 3010 Australia; ^5^ Department of Microbiology and Immunology Doherty Institute University of Melbourne Parkville Victoria 3010 Australia

**Keywords:** autophagy, bacterial membrane vesicles, bacterial pathogenesis, DNA, innate immunity, NOD2, peptidoglycan, RNA, *Staphylococcus aureus*, TLRs

## Abstract

Gram‐positive bacteria ubiquitously produce membrane vesicles (MVs), and although they contribute to biological functions, our knowledge regarding their composition and immunogenicity remains limited. Here we examine the morphology, contents and immunostimulatory functions of MVs produced by three *Staphylococcus aureus* strains; a methicillin resistant clinical isolate, a methicillin sensitive clinical isolate and a laboratory‐adapted strain. We observed differences in the number and morphology of MVs produced by each strain and showed that they contain microbe‐associated molecular patterns (MAMPs) including protein, nucleic acids and peptidoglycan. Analysis of MV‐derived RNA indicated the presence of small RNA (sRNA). Furthermore, we detected variability in the amount and composition of protein, nucleic acid and peptidoglycan cargo carried by MVs from each *S. aureus* strain. *S. aureus* MVs activated Toll‐like receptor (TLR) 2, 7, 8, 9 and nucleotide‐binding oligomerization domain containing protein 2 (NOD2) signalling and promoted cytokine and chemokine release by epithelial cells, thus identifying that MV‐associated MAMPs including DNA, RNA and peptidoglycan are detected by pattern recognition receptors (PRRs). Moreover, *S. aureus* MVs induced the formation of and colocalized with autophagosomes in epithelial cells, while inhibition of lysosomal acidification using bafilomycin A1 resulted in accumulation of autophagosomal puncta that colocalized with MVs, revealing the ability of the host to degrade MVs via autophagy. This study reveals the ability of DNA, RNA and peptidoglycan associated with MVs to activate PRRs in host epithelial cells, and their intracellular degradation via autophagy. These findings advance our understanding of the immunostimulatory roles of Gram‐positive bacterial MVs in mediating pathogenesis, and their intracellular fate within the host.

## 1 INTRODUCTION

Bacterial membrane vesicles (BMVs) are ubiquitously produced by bacteria throughout all stages of growth and are identified as a *bona fide* secretion system for the long‐distance delivery of bacterial molecules that can affect functions in host‐pathogen interactions and inter‐bacterial communication (reviewed in (Zavan et al., 2020)). Owing to the differences in the cell envelope of their parent bacteria, membrane vesicles produced by Gram‐negative bacteria which possess an outer membrane are termed outer membrane vesicles (OMVs), while those produced by Gram‐positive bacteria which lack an outer membrane are termed membrane vesicles (MVs) (Pathirana & Kaparakis‐Liaskos, 2016). The production, properties and functions of OMVs have been extensively characterized since they were first observed more than 50 years ago (Chatterjee & Das, 1967; Kaparakis‐Liaskos & Ferrero, 2015). Conversely, it was thought that the thick, rigid layer of peptidoglycan comprising the Gram‐positive cell wall prohibited the production of MVs from Gram‐positive bacteria (Lee et al., 2009). Evidence within the last decade demonstrates that MV production does occur and is broadly conserved among Gram‐positive bacteria (Brown et al., 2015; Lee et al., 2009). However, in comparison to OMVs, our understanding of the biogenesis, composition and biological functions of MVs produced by Gram‐positive bacteria is still in its infancy.

There is substantial evidence suggesting the clinical importance of OMVs produced by Gram‐negative bacteria in driving bacterial pathogenesis and disease in the host. For example, OMVs have been shown to facilitate the survival of Gram‐negative pathogens in the presence of antibiotics and to mediate the spread of antibiotic resistance through the delivery of resistance genes and antibiotic degrading enzymes to other bacteria (Rumbo et al., 2011; Stentz et al., 2015). Moreover, OMVs can enter host epithelial cells whereby they deliver microbe‐associated molecular patterns (MAMPs) to intracellular immune receptors to modulate the host immune response (Cecil et al., 2016; Kaparakis et al., 2010; Vanaja et al., 2016). OMVs have also been identified in patient tissues and biological fluids including cerebral spinal fluid, suggesting they can cross the blood‐brain barrier (Fiocca et al., 1999; Namork & Brandtzaeg, 2002). In contrast, the role of MVs in Gram‐positive bacterial pathogenesis is poorly defined. However, a growing number of studies suggest that MVs can also facilitate biological functions in inter‐bacterial communication (He et al., 2017; Liao et al., 2014) and host‐microbe interactions (Jiang et al., 2014; Tartaglia et al., 2018) in a similar manner to OMVs produced by Gram‐negative bacteria. For example, proteomic analysis of MVs has identified that they contain proteins involved in virulence (Rivera et al., 2010) and antibiotic resistance (Lee et al., 2013) that can function to mediate pathogenesis and bacterial survival in the host. Furthermore, lipoprotein delivered to host cells via MVs has been shown to drive inflammation through the activation of Toll‐like receptor 2 (TLR2) (Kim et al., 2012) and the NLRP3 (NOD‐, LRR‐ and pyrin domain‐containing protein 3) inflammasome (Wang et al., 2020). However, in contrast to our knowledge regarding OMVs, it has not been elucidated whether Gram‐positive bacteria package peptidoglycan into MVs, and only limited studies show that DNA and RNA is associated with MVs (Liao et al., 2014; Resch et al., 2016; Rodriguez & Kuehn, 2020). As a result, the ability of MVs to deliver DNA, RNA and peptidoglycan into epithelial cells and mediate activation of intracellular host pattern recognition receptors (PRRs) remains unknown. Therefore, to understand how MVs contribute to bacterial pathogenesis and disease, it is important to characterize their immunostimulatory cargo and to elucidate their ability to activate host PRRs, that will ultimately result in the induction of an innate immune response in the host.

*Staphylococcus aureus* is a major human pathogen that causes nosocomial infections with high rates of antibiotic resistance (Howden et al., 2010). MVs produced by *S. aureus* are reported to induce pro‐inflammatory cytokine production and cell death, suggesting they contribute to *S. aureus* pathogenesis and disease in the host (Tartaglia et al., 2018; Wang et al., 2020). However, as the field of MVs is still in its infancy, there is limited understanding of the MAMPs carried by *S. aureus* MVs and their ability to activate host innate immune receptors. Determining the immunogenic cargo carried by MVs and the functions they serve in host‐pathogen interactions provides insight into the mechanisms by which bacteria use MVs to facilitate host colonization and cause disease, and may uncover potential therapeutic avenues to inhibit MV production to enhance the treatment of bacterial infections (Kosgodage et al., 2019). Furthermore, understanding the mechanisms whereby MVs mediate an immune response may facilitate their development as vaccines, in a similar manner to the application of OMVs produced by Gram‐negative bacteria (Chen et al., 2010). In this study, we characterized the production, contents and innate immune effects of three *S. aureus* isolates that are known to vary in virulence; a methicillin‐resistant *S. aureus* (MRSA) clinical isolate, a methicillin‐sensitive *S. aureus* (MSSA) clinical isolate and the penicillin‐sensitive lab‐adapted strain NCTC 6571 (Heatley, 1944). The clinical isolates chosen in this study were isolated from blood cultures from confirmed *S. aureus* bacteraemia cases (Holmes et al., 2018). In contrast, NCTC 6571 lacks enzymes required for effective host colonization such as coagulase and is regarded as having low virulence (Kearns, 2006). We showed that *S. aureus* MVs produced by all strains varied in their amount and composition of nucleic acid, protein and peptidoglycan. Furthermore, we identified that DNA, RNA and peptidoglycan associated with MVs were detected by Toll‐like receptors (TLRs) and nucleotide‐binding oligomerization domain containing 2 protein (NOD2), ultimately resulting in nuclear factor‐κB (NF‐κB) activation and the production of pro‐inflammatory cytokines and chemokines by epithelial cells. Moreover, we demonstrated that internalization of *S. aureus* MVs by lung epithelial cells led to the formation of and their colocalization with autophagosomes and their subsequent degradation via the host cellular degradation pathway of autophagy. Collectively, this study identifies the ability of DNA, RNA and peptidoglycan associated with MVs to drive inflammation in *S. aureus* infections and reveals a previously undefined role of Gram‐positive bacterial MVs in inducing the cellular degradation pathway of autophagy. These findings advance our understanding of the intracellular fate of MVs produced by Gram‐positive bacteria and the immunostimulatory functions of their DNA, RNA and peptidoglycan cargo that can contribute to mediating bacterial pathogenesis and disease in the host.

## 2 MATERIALS AND METHODS

### 2.1 Bacterial culture and BMV isolation

The *S. aureus* laboratory adapted strain NCTC 6571 (Heatley, 1944) and clinical isolates BPH 2760 (VAN‐048) and BPH 2900 (VAN‐158) (Holmes et al., 2018) were maintained at 37°C on Horse Blood Agar (HBA) comprised of 8% (v/v) horse blood (Australian Ethical Biologicals, Australia) in Blood Agar Base No. 2 (Oxoid), or cultured using Brain Heart Infusion (BHI) broth (Becton Dickinson, USA) with shaking. *Helicobacter pylori* 251 Δ*cag*PAI was routinely cultured on HBA supplemented with 0.2% Skirrow's selective supplement (0.0155% (w/v) polymyxin B, 0.625% (w/v) vancomycin, 0.3125% (w/v) trimethoprim and 0.125% (w/v) amphotericin B 12.5% (w/v); Sigma). *H. pylori* was cultured at 37°C in microaerophilic conditions generated using a CampyGen 2.5L sachet (Oxoid, USA) in an anaerobic jar, as previously described (Kaparakis et al., 2010).

*S. aureus* MVs were isolated from liquid cultures that had been inoculated using overnight cultures to a starting O.D. _600nm_ of 0.1 in a volume of 200 ml BHI broth and grown at 37°C with shaking at 180 rpm for 16 h to stationary phase of growth (O.D. _600nm_ > 2.5, viable count 10^9^ colony‐forming units (CFU) per ml). *H. pylori* OMVs were isolated from liquid cultures that were inoculated using a single plate of *H. pylori* bacteria in a volume of 200 ml to achieve a starting viable count of 10^5^ CFU/ml and grown for 16 h to mid‐log phase (viable count = 10^7^ CFU/ml) in BHI supplemented with Skirrow's selective supplement and 0.6% (w/v) β‐cyclodextrin (Sigma‐Aldrich, USA) at 37°C in microaerophilic conditions with shaking at 120 rpm. To isolate either OMVs or MVs, bacteria were pelleted by centrifugation at 2500 × *g* for 30 min. Supernatants were then filtered through a 0.22 μm PES filter and MVs or OMVs were pelleted by ultracentrifugation at 100,000 × *g* for 2 h at 4°C in Ultra‐Clear 25 × 89 mm tubes (Beckman Coulter, Australia) using a P28S rotor and Hitachi CP100NX Ultracentrifuge (Hitachi, Japan). The resulting crude OMV or MV pellets were stored at ‐80°C, until further purified. Viable counts of *S. aureus* bacteria at the point of MV isolation were performed by serial dilutions that were spread onto HBA, incubated overnight at 37°C and the resulting colonies enumerated. For the purpose of facilitating transparent reporting and centralizing knowledge in extracellular vesicle research, we have submitted all relevant data of our experiments to the EV‐TRACK knowledgebase (EV‐TRACK ID: EV200185).

### 2.2 OptiPrep gradient purification of MVs

Crude *S. aureus* MVs were subsequently purified using OptiPrep (iodixanol) density gradient medium (Sigma‐Aldrich, USA), as previously described (Zavan et al., 2019). Briefly, crude MV preparations were resuspended in 2 ml PBS buffer containing 45% (v/v) OptiPrep and overlaid with 2 ml of Dulbecco's PBS (DPBS; Gibco, USA) containing 40%, 35%, 30%, 25% and 20% (v/v) OptiPrep in an Ultra‐Clear 14 × 95 mm tube (Beckman Coulter, Australia). Separation was achieved by ultracentrifugation at 100,000 x g for 16 h at 4°C using a SW40Ti rotor in an Optima L‐80XP ultracentrifuge (Beckman Coulter, Australia). Twelve 1 ml fractions were collected from the top of the density gradient. Fractions were washed twice with DPBS by ultracentrifugation at 100,000 × *g* for 2 h and resuspended in DPBS. MV‐containing fractions were identified by transmission electron microscopy for each strain, with fractions containing any contaminants excluded. Fractions 2–9 were identified as free of contaminants and pooled, re‐suspended in DPBS and pelleted by ultracentrifugation at 100,000 × *g* for 2 h at 4°C in Ultra‐Clear 25 × 89 mm tubes using a P28S rotor and Hitachi CP100NX Ultracentrifuge and the resulting MV pellet was stored at ‐80°C. All subsequent purified MV preparations were monitored by TEM for the presence of contaminants.

### 2.3 Examination of the quantity and size distribution of MVs

The quantity and size distribution of MVs were examined by nanoparticle tracking analysis (NTA) using NanoSight NS300 (Malvern Instruments, UK) and ZetaView Basic PMX‐120 (Particle Metrix, Germany). NanoSight NTA was performed as previously described (Turner et al., 2018). Briefly, purified MVs (normalized to culture volume) were diluted in DPBS to achieve a final concentration of 10^7^–10^9^ particles per ml, or 20–100 particles per field of view, as per the manufacturer's guidelines. Reads of 60 s duration were performed in triplicate at 25°C at a flow rate of 50. Capture settings were set to camera gain of 12, camera level 10, slider shutter 1300 and slider gain 512. Analysis settings were set to a detection threshold of 5, with minimum particle size, blur and minimum track length set automatically. Data outputs were generated using NanoSight NS300 NTA Software (Malvern Instruments). The average number of particles at each binned centre in the Experiment Summary output was adjusted by the dilution factor using Microsoft Excel for Office 365 MSO, and the mean of 3 biological replicates was plotted as particle size versus number of particles per ml, ± standard error of the mean (SEM) using GraphPad Prism v8.4.0 Software (GraphPad Software, USA).

ZetaView was performed using MVs diluted in DPBS. Instrument calibration was conducted prior to MV analysis using 102 nm polystyrene beads (ThermoFisher, USA), according to the manufacturer's instructions. Measurements were performed using a 405 nm 68 mW laser and CMOS camera by scanning 11 cell positions and capturing 60 frames per position at 25°C with camera sensitivity 80, shutter speed 100, autofocus and automatic scattering intensity. Analysis was performed using ZetaView Software version 8.05.12 SP1 with a minimum brightness of 30, maximum brightness 255, minimum area 5, maximum area 1000, and minimum trace length 15. The average of 3 biological replicates was plotted as particle size versus number of particles per ml using GraphPad Prism v8.4.0.

### 2.4 Transmission electron microscopy

TEM sample preparation was performed as previously described (Bitto et al., 2017). Briefly, MV samples were coated onto carbon‐coated 400 mesh copper grids (ProSciTech, Australia) by placing TEM grids on top of a 10 μl droplet of MVs at a concentration of 10^10^ MVs per ml for 10 min. Samples were subsequently fixed in 1% (w/v) glutaraldehyde (Sigma, USA) in PBS for 5 min and stained with 2% (w/v) uranyl acetate (ProSciTech, Australia) pH 7.0 for 5 min and coated with 2% (w/v) methyl‐cellulose (Sigma, USA) in 0.4% (w/v) uranyl acetate pH 4.0 for 10 min. Samples were air dried and viewed using a JEOL JEM‐2010 transmission electron microscope (JEOL, Japan) operated at 200 kV fitted with a Valeta 4 MP CCD camera (Emsis, Germany).

### 2.5 Quantification of vesicle‐associated protein, DNA, RNA and peptidoglycan

Qubit HS DNA Assay, Qubit HS RNA Assay and Qubit Protein Assay kits (Thermo Fisher Scientific, USA) were used to quantify vesicle‐associated DNA, RNA and protein respectively, using a Qubit 3.0 Fluorometer (Thermo Fisher Scientific, USA), according to manufacturer's guidelines. Peptidoglycan was quantified using a colorimetric assay based on the concentration of the muramic acid moiety, as described (Hadžija, 1974), and as previously used to detect peptidoglycan in OMVs (Kaparakis et al., 2010). Briefly, MV preparations and muramic acid standard solutions, prepared from L‐18 muramyldipeptide (Invivogen, USA), were diluted in 0.5 ml of 1M NaOH. Samples were incubated at 38°C for 30 min prior to the addition of 0.5 ml 0.5 M H_2_SO_4_ and 5 ml concentrated H_2_SO_4_. Solutions were boiled for 5 min, then cooled under running water, followed by the addition of 0.05 ml CuSO_4_ (4% w/v) and 0.1 ml of 1.5% (w/v) 4‐phenylphenol dissolved in 96% ethanol. Samples were incubated at 30°C for 30 min and the absorbance was measured at 560 nm using a spectrophotometer.

### 2.6 SDS‐PAGE and Western immunoblot

*S. aureus* whole cell lysates for separation of proteins by SDS‐PAGE were prepared by sonicating bacteria for 3×30 sec intervals on ice after incubation with 1 mg/ml lysostaphin (Sigma Aldrich, USA) in TE buffer at 37°C for 30 min. *S. aureus* whole cell lysates for separation of peptidoglycan by SDS‐PAGE were prepared by sonicating for 10 × 30 s intervals on ice in TE buffer. Whole cell lysates and MVs (normalized to protein amount or MV number, as indicated) were boiled for 5 min in 1x NuPAGE LDS sample buffer (ThermoFisher, USA) containing 1 × NuPAGE Reducing Agent (ThermoFisher, USA) and separated using a Bolt 4%–12% Bis‐Tris SDS PAGE (Life Technologies, USA) gel with NuPAGE MES SDS running buffer (Life Technologies, USA) at 80 volts for 2 h. Proteins were transferred onto a polyvinylidene difluoride (PVDF) membrane using a wet transfer system at 30 V for 45 min. Membranes were blocked with 1% (w/v) bovine serum albumin (BSA) in Tris‐buffered saline with 0.05% Tween20 (TBS‐T) for 2 h at room temperature (RT), followed by incubation overnight at 4°C with rabbit polyclonal anti‐*S. aureus* IgG antibody (Bio‐Rad Laboratories, USA; product code 0300‐0084) or mouse monoclonal anti‐peptidoglycan antibody (Bio‐Rad Laboratories, USA; clone number 3F6B3) (Warnking et al., 2015), diluted 1:1000 in 1% BSA in TBS‐T. Membranes were then washed in TBS‐T and incubated for 1 h at RT with goat anti‐rabbit secondary antibody conjugated with horseradish peroxidase (ThermoFisher, USA) or goat anti‐mouse secondary antibody conjugated with horseradish peroxidase (ThermoFisher, USA), diluted 1:5000 in 1% BSA in TBS‐T. Membranes were washed in TBS‐T, developed using Clarity Western ECL Substrate (Bio‐Rad Laboratories, USA) and imaged using a GE Amersham Imager 600 (GE Life Sciences, UK).

### 2.7 RNA extraction and analysis

RNA was isolated from *S. aureus* MVs using the miRNeasy Mini Kit (Qiagen, Germany). The composition and quality of purified RNA was analyzed using the Agilent 2100 Bioanalyzer for small RNA profiles with the Small RNA kit (Agilent, Tokyo), as previously described (Cheng et al., 2014). Isolated RNA was comprised of mostly small RNA <100 nt in length.

### 2.8 Cell culture and stimulations

Lung epithelial A549 cells were maintained in Roswell Park Memorial Institute (RPMI) media (Gibco, USA) supplemented with 10% (v/v) fetal bovine serum (Gibco, USA), 1% (v/v) L‐glutamine (Gibco, USA) and 1% (v/v) penicillin/streptomycin (Gibco, USA). A549 cells were seeded at 2×10^5^ cells/ml in 500 μl in 24‐well plates and stimulated with MVs for 24 h in serum‐free RPMI. *S. aureus* were grown to an O.D. _600nm_ of 0.5, washed with PBS and added to A549 cells in antibiotic‐free RPMI at a MOI of 100 bacteria per cell. Cells were co‐cultured with bacteria for 2 h then media was replaced with fresh RPMI containing 100 μg/ml gentamicin (Sigma, USA), 20 μg/ml lysostaphin (Sigma, USA) and 1% (v/v) penicillin/streptomycin (Gibco, USA) as previously described (Yang & Ji, 2014). After 24 h, cell culture supernatants were removed and stored at ‐80°C for further analysis by cytometric bead array (CBA) immunoassay.

HEK‐Blue cell lines Null, hTLR2, hTLR4, hTLR7, hTLR8, hTLR9 and hNOD2 containing an inducible NF‐κB/AP1 secreted alkaline phosphatase (SEAP) reporter (InvivoGen, USA) were maintained as previously described (Cecil et al., 2016). Briefly, cells were grown in Dulbecco's Modified Eagle Media (DMEM; Gibco, USA) supplemented with 10% foetal bovine serum (Gibco, USA), 1% L‐glutamine (Gibco, USA) and 1% penicillin/streptomycin (Gibco, USA) with selective antibiotics for each cell line: 100 μg/ml Zeocin for Null cells, 10 μg/ml Blasticidin and 100 μg/ml Zeocin for TLR7 and TLR9 cells, 30 μg/ml Blasticidin and 100 μg/ml Zeocin for TLR8 and NOD2 cells, 30 μg/ml Blasticidin and 200 μg/ml Hygromycin and 100 μg/ml Zeocin for TLR2 and TLR4 cells (all obtained from InvivoGen, USA). HEK‐Blue cells were seeded at 1 × 10^6^ cells/ml in 200 μl in 96‐well plates and stimulated for 24 h with an increasing dose of purified MVs. Positive controls for each cell line were as follows: 50 ng/ml Pam3CSK4 (Pam3CysSerLys4; InvivoGen, USA) for TLR2 cells, 6.25 ng/ml LPS (InvivoGen, USA) for TLR4 cells, 1 pg/ml R848 (resiquimod; InvivoGen, USA) for TLR7 and TLR8 cells, 5 nM CpG ODN (InvivoGen, USA) for TLR9 cells or 0.001 pg/ml L18‐MDP (InvivoGen, USA) for NOD2 cells. Cell culture supernatants were assayed for secreted alkaline phosphatase (SEAP) activity by incubating 20 μl of supernatant with 180 μl of QUANTI‐Blue solution (InvivoGen, USA) at 37°C, according to manufacturer's instructions. SEAP activity was measured at 620 nm using a FLUOstar OPTIMA plate reader (BMG Labtech, Australia).

### 2.9 Multiplexed cytometric bead array (CBA) immunoassay

Cytokine and chemokine profiling was performed using a cytometric bead array (CBA) multiplex immunoassay to detect IL‐8, CCL2 (MCP‐1) and IL‐6 (BD Biosciences, Australia) as previously described (Phan et al., 2018). All assays were performed in accordance with the manufacturer's instructions and analyzed using a BD CANTO II. Data analysis was performed using the FCAP Array v3 software.

### 2.10 Confocal microscopy

Fluorescent staining of MV‐associated DNA was performed as previously described (Bitto et al., 2017). Briefly, 1 × 10^10^ MVs were incubated with or without 2 U of TURBO DNaseI (Ambion, USA) for 20 min at 37°C, then coated onto glass coverslips using poly‐L lysine (Sigma, USA). MV‐associated DNA was labelled with SYTO‐61 (Molecular Probes, USA) at a dilution of 1:1,000 in PBS, followed by 4 × 5 min washes with PBS. Coverslips were mounted onto slides using VectaShield Mounting Medium (Vector Laboratories, USA) and imaged using a Zeiss LSM 780 PicoQuant confocal microscope (Zeiss, Germany) using a 100x/1.46NA oil objective at 1024 × 1024 × 32 bit per channel. Image processing and analysis was performed using Imaris v7.6.5 (Bitplane, Switzerland).

Autophagy experiments were performed as previously described (Irving et al., 2014). Briefly, *H. pylori* OMVs or *S. aureus* MVs were incubated with the lipophilic fluorescent dye Vybrant DiI (Molecular Probes, USA) diluted 1:100 in PBS for 30 min at 37°C. Unbound dye was removed by washing OMVs or MVs three times with 4 ml PBS using a 10 kDa MWCO Amicon filtration column. A549 cells were seeded at 5 × 10^3^ cells in 8‐well chamber slides (ThermoFisher, USA) and transfected the following day with 60 ng of light chain 3 (LC3)‐GFP fluorescent construct and 170 ng of pcDNA3 empty vector, using Lipofectamine 2000 (Life Technologies, USA) according to manufacturer's instructions. After 24 h incubation, media was replaced with serum‐free RPMI and cells were pre‐treated with 10 nM bafilomycin A1 (Sigma Aldrich, USA) or not treated as a control and then stimulated with either 100 nM rapamycin (Sigma‐Aldrich, USA), 1 × 10^10^ DiI‐labelled *H. pylori* OMVs or *S. aureus* MVs for 1, 2, 3 or 4 h. Non‐stimulated cells served as a negative control. Extracellular fluorescence was quenched using 0.025% trypan blue and cells were then washed with PBS, fixed in 4% paraformaldehyde (Sigma‐Aldrich, USA), and cellular actin and nuclei were stained with Alexa Fluor 680 phalloidin (Molecular Probes, USA) and 4’,6‐diamidino‐2‐phenylindole dilactate (DAPI; Merck, Germany) respectively, according to manufacturer's instructions. Samples were mounted in VectaShield Mounting Medium (Vector Laboratories, USA) and imaged using a Zeiss LSM 780 PicoQuant confocal microscope (Zeiss, Germany) using a 63x/1.4NA oil objective at 1024 × 1024 × 32 bit per channel.

### 2.11 Image analysis

Image analysis was performed using Imaris x64 v9.5.0 (Bitplane, Switzerland). Autophagosomes per cell were quantified as previously described (Irving et al., 2014) using Imaris Cell to detect cell boundaries and nuclei, followed by vesicle detection and thresholding based on autophagosome size (number of voxels), intensity (max intensity, intensity centre) and the built‐in ‘quality’ threshold. Colocalization analysis was performed as previously described (Irving et al., 2014) using the built‐in Imaris Coloc feature. Thresholding was performed using a reference image of the positive control and maintained for all subsequent analyses. Three biological replicates were examined, and for each replicate three separate fields of view were imaged per treatment, containing a minimum of 10 cells per field of view and a minimum of 50 cells per biological replicate.

### 2.12 Statistical analysis

Data analysis was performed using GraphPad PRISM v8.4.0. All data are presented as mean ± standard error of the mean (SEM) unless otherwise stated. Statistical analyses were performed using data from three biological replicates, in triplicate, using the One‐way ANOVA with Tukey's multiple comparisons test or Dunnett's multiple comparisons test as indicated.

## 3 RESULTS

### 3.1 *S. aureus* strains produce MVs that vary in amount and morphology

**FIGURE 1:**
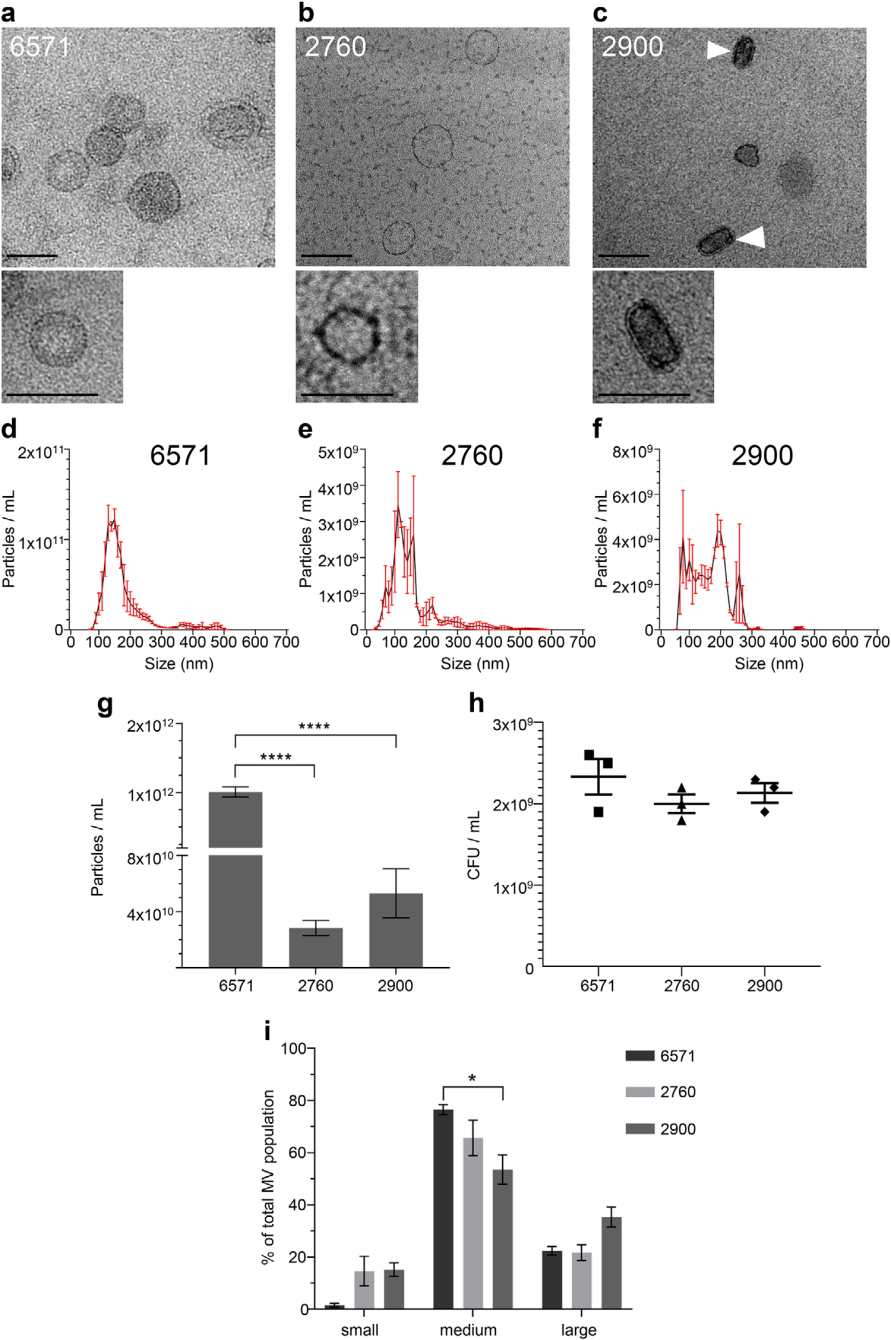
Membrane vesicles isolated from *S. aureus* strains 6571, 2760 and 2900 vary in size and quantity. Transmission electron microscopy (TEM) of (a) 6571, (b) 2760 and (c) 2900 MVs (arrows indicate elliptical MVs), showing both wide‐field images and magnified images of single MVs of each strain. TEM images are representative of three biological replicates. Scale bar = 100 nm. Size distribution of MVs isolated from (d) 6571, (e) 2760 and (f) 2900 determined by NanoSight NTA. Data show the mean of three biological replicates (black line) ± SEM (red error bars). (g) Total number of particles per ml produced by each strain as indicated, determined by NanoSight NTA. Data show mean of three biological replicates ± SEM. ****P < 0.0001 (One‐way ANOVA with Tukey's multiple comparisons test). (h) The number of viable bacteria present in individual cultures at the point of MV isolation for each strain. Data show CFU/ml of individual cultures, and the mean ± SEM of three biological replicates. (i) Quantification by NanoSight NTA of the size distribution of small (<100 nm), medium (100‐200 nm) and large (>200 nm) MVs found within heterogenous MV samples produced by *S. aureus* strains, as indicated. Data are calculated from the average of three biological replicates ± SEM. *P < 0.05 (One‐way ANOVA with Tukey's multiple comparisons test).

To determine if bacterial strain differences exist in the production, morphology and composition of *S. aureus* MVs, we isolated and examined MVs produced by three *S. aureus* strains (Holmes et al., 2018; Kearns, 2006), denoted 6571, 2760 and 2900. MVs produced by each strain were purified by OptiPrep gradient ultracentrifugation and MV‐containing fractions were identified by transmission electron microscopy (TEM; Supplementary Figure 1). Purified MVs were visualized by TEM (Figure 1(a‐c)), revealing differences in the morphology of MVs among strains, as both strains 6571 and 2760 produced MVs that were spherical (Figure 1(a‐b)), whereas 2900 produced a mixed type of MVs that were either elliptical or spherical in morphology (Figure 1(c)).

We next used NanoSight and ZetaView Nanoparticle Tracking Analysis (NTA) to further examine the quantity and size distribution of MVs produced by all strains. Both NanoSight and ZetaView identified that all strains produced MVs that were heterogeneous in size, ranging from 50–500 nm (Figure 1(d‐f), Supplementary Figure 2). Quantification of the total number of MVs produced by each strain revealed that the 6571 laboratory strain produced significantly more MVs when compared to both the 2760 and 2900 clinical strains, indicating that there are strain differences in the amount of MVs produced (Figure 1(g), *P* < 0.0001). Viable counts of the bacterial cultures at the time of MV isolation showed that there was no statistical difference in the number of viable bacteria present in the cultures of each strain, suggesting that differences in the amount of MVs produced by each strain was not growth dependent (Figure 1(h)). Examination of the size distribution of small (<100 nm), medium (100‐200 nm) and large (>200 nm) MVs produced by each strain revealed significant inter‐strain differences in the percentage of vesicles within the different size populations, suggesting that there is heterogeneity in the size distribution of MV produced by different strains (Figure 1(i)). Collectively, these results show that there is inter‐strain variability in the morphology, amount and size distribution of MVs produced by *S. aureus* strains.

**FIGURE 2:**
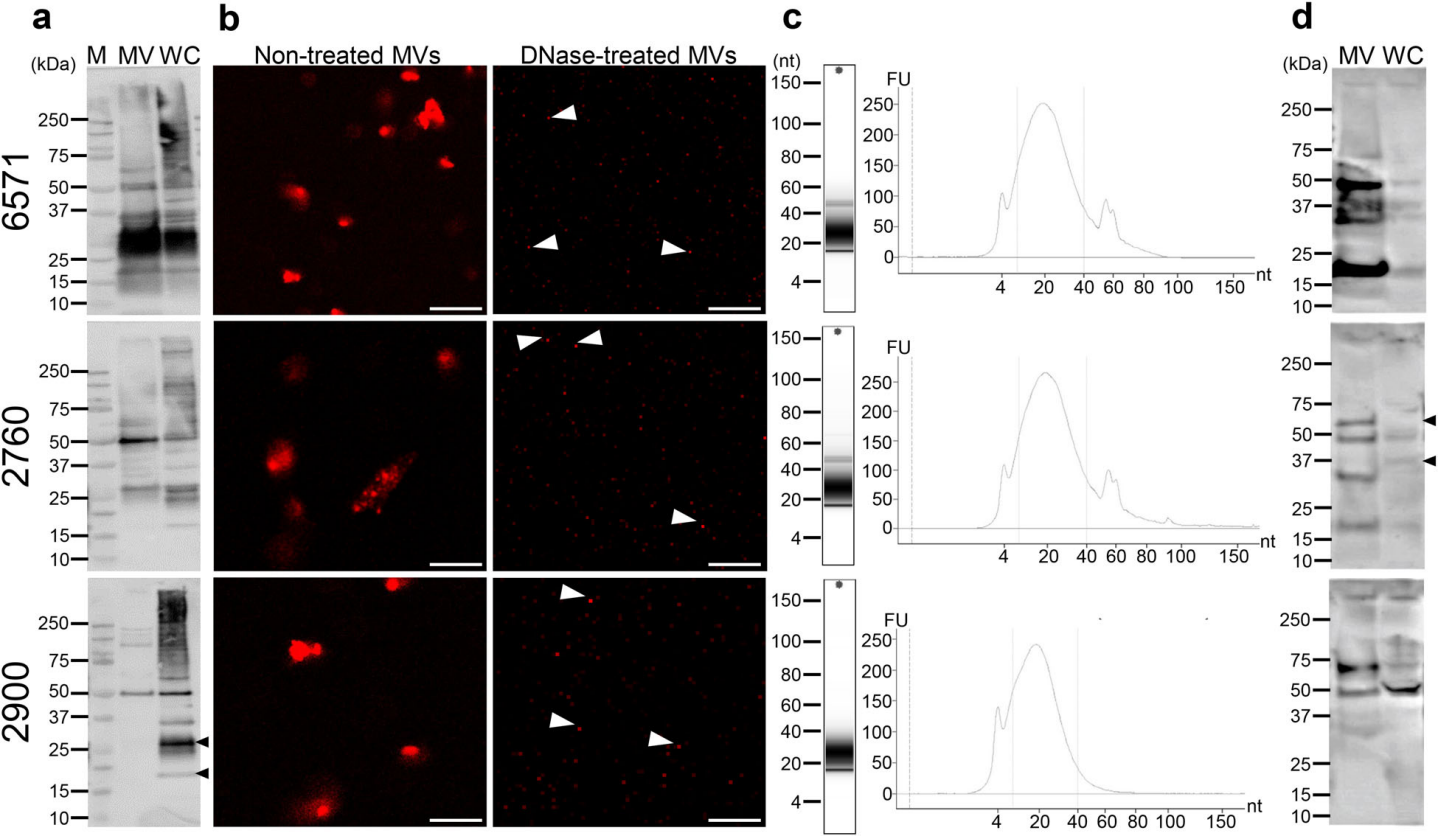
Detection of the protein, DNA, RNA and peptidoglycan cargo of *S. aureus* MVs. Characterization of MV contents from *S. aureus* strains 6571, 2760 and 2900, as indicated. (a) Protein content of MVs and their parent bacteria was examined using Western immunoblot. MVs (MV; 10 μg) and whole‐cell bacterial lysate (WC; 10 μg) proteins were detected using anti‐*S. aureus* polyclonal antibodies. The black arrows highlight differences in the protein content of 2900 WC compared to 2900 MVs. M = Precision Plus Protein standard (Bio‐Rad Laboratories). (b) Confocal microscopy was used to visualize the location and amount of DNA associated with MVs. MVs were treated with or without DNase and stained with the membrane permeable DNA stain SYTO‐61, showing both external DNA (non‐treated MVs) and internal DNA (DNase‐treated MVs) as indicated. Arrows highlight internal DNA remaining after DNase‐treatment. Scale bar = 2 μm. Representative of n = 3 biological replicates. (c) RNA was extracted from 6571, 2760 and 2900 MVs and analyzed using the Agilent Bioanalyzer. Tapestation gel shows RNA profile and graph shows RNA concentration in fluorescent units (FU) vs. nucleotide length (nt). The size range of small RNAs (sRNA) of 10 – 40 nt is indicated by the dotted lines. Representative of n = 3 biological replicates. (d) Detection of MV‐associated peptidoglycan by Western immunoblot. MVs (MV; 10 μg) and whole‐cell bacterial lysate (WC; 10 μg) were separated by SDS‐PAGE and peptidoglycan was detected using an anti‐peptidoglycan antibody. The black arrows highlight differences in the peptidoglycan content of 2760 WC compared to 2760 MVs.

### 3.2 *S. aureus* MVs produced by different strains vary in their protein, nucleic acid and peptidoglycan quantity and composition

OMVs produced by Gram‐negative bacteria are known to contain highly diverse contents that can stimulate a range of host PRRs, including protein, DNA, RNA and peptidoglycan, and this content can vary significantly between strains (Avila‐Calderón et al., 2012; Bielaszewska et al., 2017; Ferrer‐Navarro et al., 2016; Han et al., 2019; Irving et al., 2014; Kaparakis et al., 2010; Li et al., 2015; Perez Vidakovics et al., 2010). In contrast, there is relatively little known about the composition of MVs produced by Gram‐positive bacteria, in particular their nucleic acid and peptidoglycan content, and the ability of these components to activate host PRRs (Choi et al., 2018; Lee et al., 2009; Liao et al., 2014; Rivera et al., 2010; Rodriguez & Kuehn, 2020). To address this, we compared and quantified the protein, DNA, RNA and peptidoglycan content of *S. aureus* MVs from the lab adapted strain 6571 and clinical isolates 2760 and 2900.

MV‐associated proteins were detected by Western immunoblot using a polyclonal anti‐*S. aureus* antibody that reacts with soluble and structural antigens of the whole bacterium, revealing that MVs contain numerous proteins of varying molecular weight (Figure 2(a); (Supplementary Figure 3)). Comparison of the overall protein composition of MVs between strains was performed by examining equal amounts of MVs from each strain (1×10^10^) by Western immunoblot (Supplementary Figure 3(a)). This revealed that there were differences in the broad protein composition of MVs between strains. Some MVs were enriched in proteins of a particular size compared to MVs produced by other strains, such as bands at approximately 125, 100 and 45 kDa present predominantly in 2760 MVs (Supplementary Figure 3(a), black arrows) and bands of approximately 28, 25 and 18 kDa seen in 6571 and 2760 MVs but not 2900 MVs (Supplementary Figure 3(a), black diamonds). Furthermore, some predominant protein bands present in bacteria did not appear to be equally enriched in MVs, such as bands of approximately 25 and 20 kDa present in 2900 bacteria but not their MVs (Figure 2(a), black arrows). These findings suggest that there may be preferential packaging of protein cargo into MVs, and is consistent with our previous findings identifying selective cargo packaging of proteins into OMVs produced by Gram‐negative bacteria (Zavan et al., 2019).

Studies have shown that OMVs carry DNA bound to the surface and within the lumen (Bitto et al., 2017; Pérez‐Cruz et al., 2013), however it is unclear whether this is also the case for MVs. To address this, MVs were treated with or without DNase, which can degrade DNA on the surface of MVs but not that which is protected within the vesicle membrane (Bitto et al., 2017). MVs were then stained using the membrane‐permeable DNA stain SYTO‐61 and visualized by confocal microscopy (Figure 2(b)). DNA was detected on the surface of 6571, 2760 and 2900 MVs (Figure 2(b) ‘Non‐treated’). A significant reduction in the amount of DNA associated with MVs was noted in all DNase‐treated MVs samples (Figure 2(b) ‘DNase‐treated’), compared to their non‐DNase treated controls, suggesting that DNA associated with *S. aureus* MVs is predominantly bound to the exterior MV surface with minimal DNA being contained and protected within MVs.

RNA secretion is a newly recognized area of bacterial communication and host‐pathogen interactions (Lee, 2019). The RNA content of OMVs has recently gained interest, with particular focus on bacterial small RNA (sRNA) species that may have a potential role in transcriptional regulation in target cells (Koeppen et al., 2016). However, there are still very limited studies that characterize the RNA cargo of MVs produced by Gram‐positive bacteria (Choi et al., 2017; Joshi et al., 2021; Lee & Hong, 2012; Rodriguez & Kuehn, 2020). To address this, we isolated sRNA from *S. aureus* MVs produced by all 3 strains and assessed the RNA profile using an Agilent Bioanalyzer instrument with an Agilent Small RNA Kit, which analyzes the yield of sRNA ranging between 4–200 nt (Figure 2(c)). The data showed sRNA of less than 100 nucleotides (nt) in length was associated with MVs produced by all *S. aureus* strains (Figure 2(c)), and that all samples contained a predominant RNA peak at 10–40 nt (Figure 2(c)), which is consistent with the size of bacterial sRNA (Lee & Hong, 2012). There was also a smaller peak of RNA approximately 60 nt in size present in the 6571 and 2760 MVs that was absent from the 2900 strain, identifying inter‐strain differences in MV RNA composition. This data shows the presence of sRNA associated with *S. aureus* MVs produced by multiple strains, that may play a role in host‐pathogen interactions and inter‐bacterial communication.

We have previously identified the presence of peptidoglycan in OMVs produced by Gram‐negative bacteria and their delivery to the host cell cytoplasm, whereby they activate the cytosolic receptor NOD1 and induce the cellular degradation pathway of autophagy. In contrast, there are no reports identifying the presence of peptidoglycan within MVs produced by Gram‐positive bacteria, and hence their role in activating innate immune receptors that are responsible for the detection of peptidoglycan is unknown. To address this, equal amounts of MVs from each *S. aureus* strain were separated by SDS‐PAGE and peptidoglycan was detected by Western immunoblot using an anti‐peptidoglycan antibody (Figure 2(d), Supplementary Figure 3(b)). A range of different sized muropeptide bands were detected in MVs, which is consistent with previous detection of *S. aureus* peptidoglycan by Western immunoblot using the same antibody (Warnking et al., 2015). Interestingly, differences in muropeptide bands were observed between MVs and the whole‐cell lysate of their parent bacteria, such as a band at approximately 60 kDa present in 2760 MVs but not the bacterial lysate, and a 37 kDa band present in 2760 bacterial lysate but not MVs (Figure 2(d), black arrows), which suggests possible modification of peptidoglycan moieties during MV formation. In addition, differences were observed in the muropeptide bands between MVs from each strain normalized to particle number (Supplementary Figure 3(b)), such as a band of approximately 75 kDa present in 6571 and 2900 MVs but not in 2760 MVs, and a band at approximately 18 kDa present in 6571 and 2760 MVs but not in 2900 MVs. These differences suggest that there may be strain differences in the tertiary structure, cross‐bridging or acetylation of peptidoglycan carried by MVs.

**FIGURE 3:**
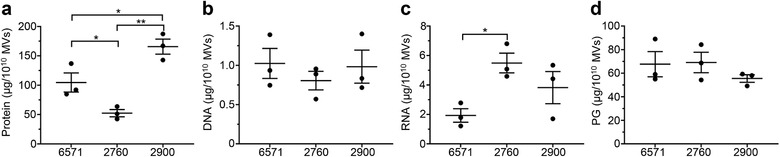
Quantification of the protein, DNA, RNA and peptidoglycan cargo of *S. aureus* MVs. Quantification of (a) protein, (b) DNA, (c) RNA and (d) peptidoglycan (PG) associated with MVs from *S. aureus* strains 6571, 2760 and 2900. Protein, DNA and RNA were quantified by Qubit, while peptidoglycan was quantified colorimetrically based on the concentration of muramic acid present in the MVs. Data shows individual biological MV samples and indicates the mean ± SEM of three biological replicates. *P < 0.05, **P < 0.01 (One‐way ANOVA with Tukey's or Dunnett's multiple comparisons tests).

The amount of protein, DNA, RNA and peptidoglycan associated with 6571, 2760 and 2900 MVs was quantified using Qubit, while peptidoglycan was quantified using a colorimetric assay based on the concentration of muramic acid present in the MV preparations (Figure 3(a‐d)). Concentrations were normalized as micrograms per 1 × 10^10^ MVs to account for the differences in the quantity of MVs produced by each strain (Figure 3). We found that while the quantity of DNA and peptidoglycan associated with MVs was consistent between strains (Figure 3(b,d)), the protein and RNA content of MVs differed significantly (Figure 3(a,c)). In particular, MVs from the 2760 strain contained significantly less protein when compared to MVs from all other strains (Figure 3(a)), in addition to containing significantly more RNA when compared to the lab adapted 6571 strain (Figure 3(c)).

### 3.3 *S. aureus* MVs containing DNA, RNA and peptidoglycan are detected by TLRs and NOD2 innate immune receptors resulting in NF‐κB activation

**FIGURE 4:**
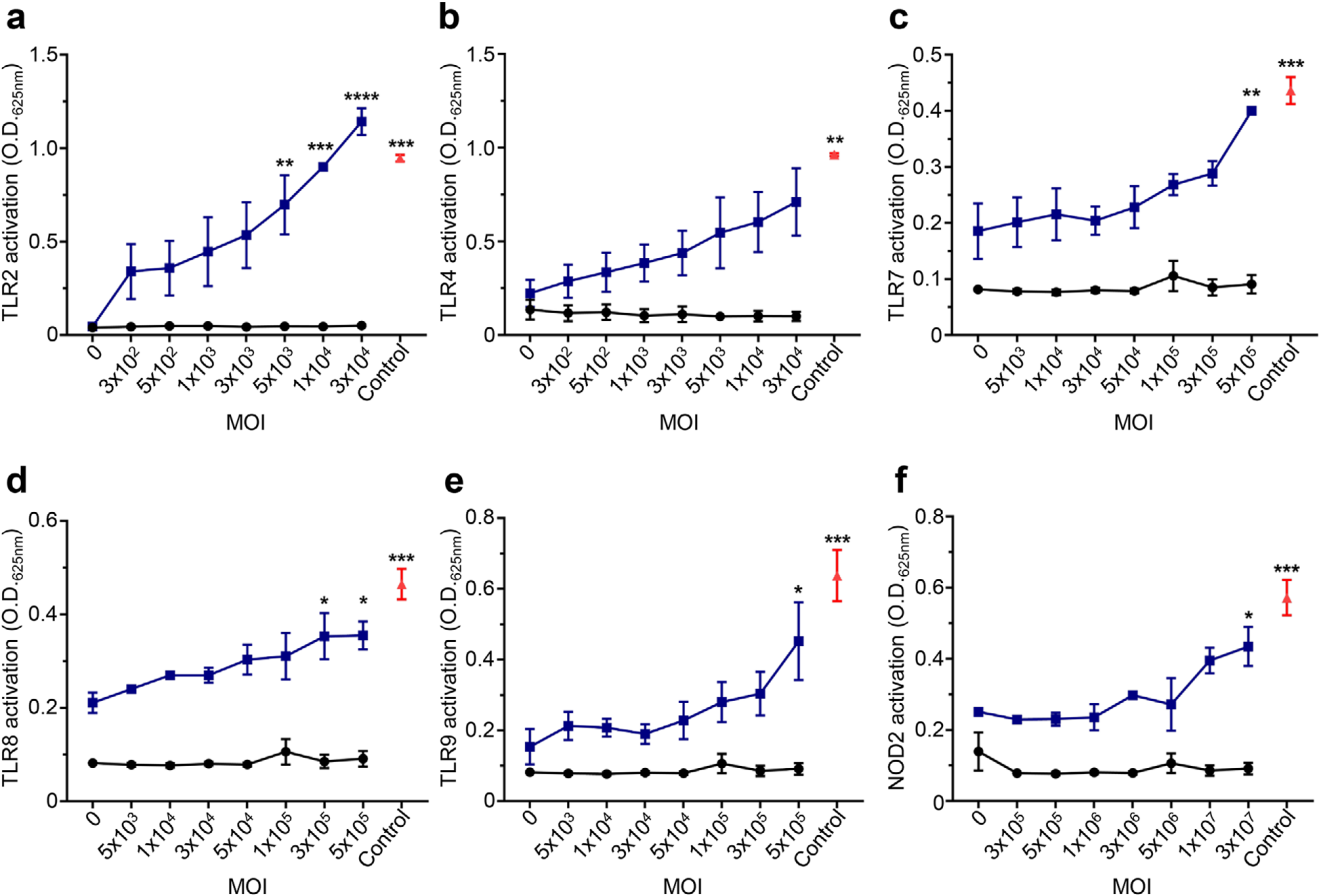
DNA, RNA and peptidoglycan associated with *S. aureus* MVs activate TLR and NOD2 signaling. The ability of 6571 MVs to activate specific PRRs was investigated using HEK‐Blue reporter cell lines expressing (a) TLR2, (b) TLR4, (c) TLR7, (d) TLR8 or (e) TLR9, or (f) NOD2 receptors (blue). Cells were stimulated with an increasing MOI of 6571 MV (x‐axis). Positive controls for each respective cell line (red) include 50 ng/ml Pam3CSK4 (TLR2), 6.25 ng/ml LPS (TLR4) 1 pg/ml R848 (TLR7 and TLR8), 5 nM CpG ODN (TLR9) or 0.001 pg/ml L18‐MDP (NOD2). HEK‐Blue Null cells were used as a negative control (black). Data are represented as mean ± SEM of three biological replicates. Significance was calculated by comparing to the non‐stimulated control of the same cell line. *P < 0.05, **P < 0.01, ***P < 0.001, ****P < 0.0001 (One‐way ANOVA with Dunnett's multiple comparisons test).

Numerous studies have reported the ability of OMV components including lipopolysaccharide (LPS), peptidoglycan and nucleic acids to activate PRRs such as membrane‐bound Toll‐like receptors (TLRs) and the cytoplasmic nucleotide‐binding oligomerization domain containing protein (NOD) receptors (Bielig et al., 2011; Han et al., 2019; Kaparakis et al., 2010; Marion et al., 2019). However, in comparison to OMVs, our understanding of the roles MVs serve in host‐pathogen interactions is limited, and the ability of MVs to activate TLRs and NOD receptors via their RNA, DNA and peptidoglycan content remains poorly understood. In particular, it is not known whether MVs transport their RNA, DNA and peptidoglycan cargo into the cytoplasm of non‐phagocytic epithelial cells where they can subsequently be detected by and activate intracellular innate immune receptors. To address this, we investigated the ability of *S. aureus* 6571 MVs to signal via a range of host PRRs using HEK‐Blue reporter cell lines. HEK‐Blue cells expressing either TLR2, 4, 7, 8, 9 or NOD2 receptors were stimulated with an increasing number of 6571 MVs per cell, or ‘multiplicity of infection’ (MOI). We found that *S. aureus* MVs could mediate NF‐κB activation via TLR2 (Figure 4(a)), which is known to be activated by *S. aureus* lipoteichoic acids (Kim et al., 2012) and peptidoglycan (Schwandner et al., 1999). Although TLR4 is traditionally considered a receptor for Gram‐negative bacterial lipopolysaccharide, there are a small number of reports of *S. aureus* peptidoglycan weakly activating TLR4 (Hadley et al., 2005; Yang et al., 2008), however this is yet to be definitively shown. Our data shows a positive correlation between MV concentration and NF‐κB activation in HEK‐Blue TLR4 cells, however there was no statistical significance in TLR4 mediated responses at any of the MV concentrations tested (Figure 4(b)).

As bacterial RNA and DNA is detected by TLR7, 8 and 9 respectively (Eigenbrod & Dalpke, 2015; Jurk et al., 2002; Latz et al., 2004), we next examined the ability of MV‐associated DNA and RNA to mediate NF‐κB signalling in HEK‐Blue reporter cells. Activation of the endosomal nucleic acid receptors TLR7, 8 and 9 were observed in response to stimulation with an increasing dose of MVs (Figure 4(c‐e)), revealing an immunomodulatory role for *S. aureus* MV‐associated RNA and DNA. (Jurk et al., 2002; Latz et al., 2004). In addition, the cytoplasmic receptor for Gram‐positive bacterial peptidoglycan, NOD2, was activated by *S. aureus* MVs in a dose‐dependent manner (Figure 4(f)), identifying the previously unknown ability of NOD2 to detect peptidoglycan contained within MVs produced by Gram‐positive bacteria (Girardin et al., 2003). Collectively, these findings elucidate the modulation of innate immune responses by *S. aureus* MVs and suggests that their immunostimulatory cargo can contribute to driving inflammation during *S. aureus* infection, which may potentially facilitate the disruption of the epithelial barrier to allow colonization of host tissues (Chi et al., 2003; Nakao et al., 2014).

### 3.4 *S. aureus* MVs induce pro‐inflammatory cytokine and chemokine responses in a strain‐dependent manner

**FIGURE 5:**
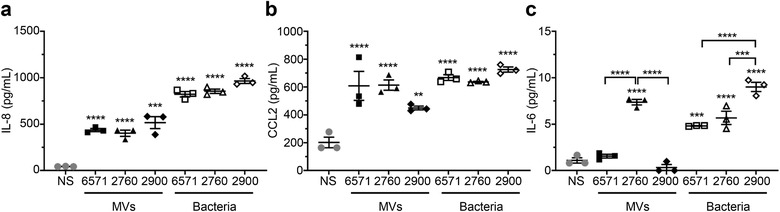
*S. aureus* MVs induce the production of pro‐inflammatory cytokines and chemokines by lung epithelial cells in a strain dependent manner. The amount of (a) IL‐8, (b) CCL2 and (c) IL‐6 produced by A549 cells in response to stimulation with 1 × 10^10^ MVs (MOI = 5 × 10^4^ MVs per cell, black filled shapes) from *S. aureus* strains 6571, 2760 and 2900, was determined using cytometric bead array (CBA) analysis. Non‐stimulated (NS, grey circles) A549 cells, or cells stimulated with *S. aureus* bacteria (open shapes) at a multiplicity of infection (MOI) of 100 were used as negative and positive controls, respectively. Data show the mean of three biological replicates ± SEM. **P < 0.01, ***P < 0.001, ****P < 0.0001 compared to the NS control, unless indicated with brackets (One‐way ANOVA with Tukey's multiple comparisons test).

As signalling of MAMPs via PRRs results in the activation of NF‐κB and ultimately the induction of a pro‐inflammatory response, we next investigated the ability of *S. aureus* 6571, 2760 and 2900 MVs to induce the production of pro‐inflammatory cytokines and chemokines in A549 human lung epithelial cells. To enable comparisons of the immunostimulatory ability of MVs from each strain, we normalized the number of MVs used in each stimulation to 1 × 10^10^ MVs, which is equivalent to a MOI of 5 × 10^4^ MVs per A549 cell. As a positive control, whole live bacteria were added to cells. Production of interleukin‐8 (IL‐8), CC‐motif ligand 2 (CCL2, also known as monocyte chemoattractant protein‐1, or MCP‐1) and interleukin‐6 (IL‐6) was detected in response to A549 cells stimulated with either 6571, 2760 or 2900 MVs or their parent bacteria (Figure 5), and although interleukin‐1β (IL‐1β) and tumour necrosis factor‐α (TNF‐α) production was examined, their levels were below the detection limit of the assay (not shown). A significant increase in IL‐8 was detected in the supernatants of A549 cells treated with either 6571, 2760 or 2900 MVs, compared to non‐stimulated cells (Figure 5(a)). However, there was no significant difference in the amount of IL‐8 produced in response to stimulation with either 6571, 2760, 2900 MVs or bacteria (Figure 5(a)). Similarly, a significant increase in CCL2 was observed in response to MVs and bacteria of each strain compared to the non‐stimulated control, with no strain‐specific differences observed (Figure 5(b)). In contrast, IL‐6 production by A549 cells was only detected in response to stimulation with 2760 MVs, despite the ability of all 3 strains of bacteria to induce IL‐6 production by these cells (Figure 5(c)). Collectively, these results demonstrate that *S. aureus* MVs mediate a pro‐inflammatory response in epithelial cells however, bacterial MV strain differences may determine the type of cytokines and chemokines produced.

### 3.5 *S. aureus* MVs induce the formation of LC3‐GFP aggregates and an autophagy response in lung epithelial cells

We have previously shown that OMVs function as a novel mechanism used by Gram‐negative bacteria to transport their peptidoglycan into the cytoplasm of the host cell, resulting in the induction of a NOD1‐mediated pro‐inflammatory response (Kaparakis et al., 2010). This in turn results in the degradation of OMVs by epithelial cells via autophagy in a NOD1‐dependent manner (Irving et al., 2014). However, to date, the ability of MVs produced by Gram‐positive bacteria to induce and be degraded by autophagy remains unknown. As we identified that *S. aureus* MVs can mediate NOD2 signalling (Figure 4(f)), we next examined whether *S. aureus* MVs mediate the induction of autophagy once they enter the cytoplasm of host epithelial cells.

**FIGURE 6:**
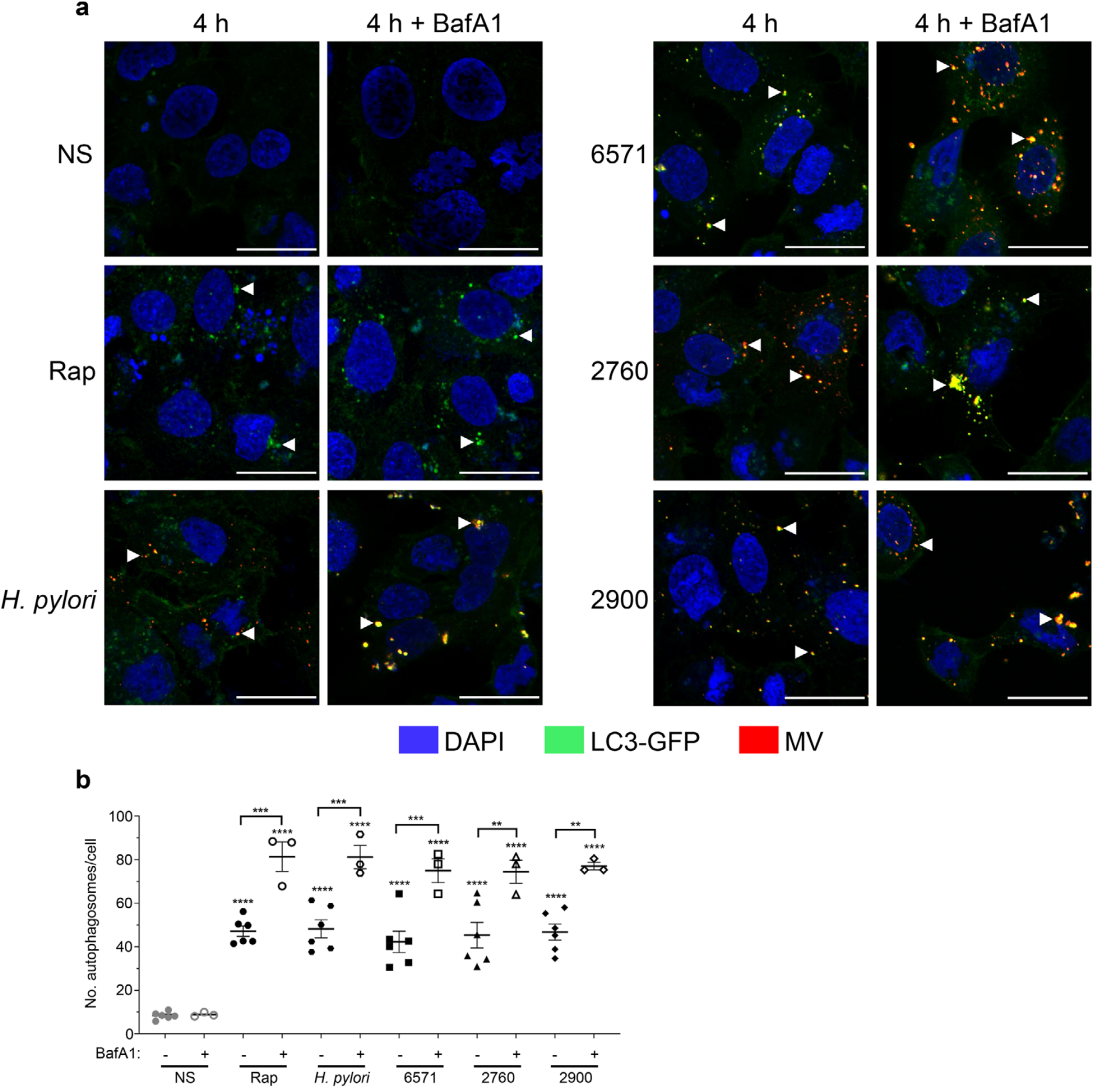
*S. aureus* MVs induce the formation of autophagosomes in lung epithelial cells. (a) A549 cells were transfected with LC3‐GFP (green) and stimulated for 4 h with DiI‐labelled 6571, 2760 or 2900 MVs (red), or positive controls DiI‐labelled *H. pylori* OMVs (red) or 100 ng/ml rapamycin (Rap). Cells were also pre‐treated with 10 nM bafilomycin A1 (BafA1), an inhibitor of phagolysosomal degradation, for 30 min prior to stimulation. Non‐stimulated cells (NS) served as a negative control. Cell nuclei were stained using DAPI (blue). Autophagosome formation is evident by the formation of LC3‐GFP puncta (white arrow heads) and colocalization of MVs with LC3‐GFP puncta is seen in yellow. Images were acquired in 3D and show z projections of the 3D image. Images are representative of three biological replicates. Scale bar = 20 μm. (b) Quantification of LC3‐GFP puncta in cells pre‐treated with or without BafA1 then stimulated for 4 h with MVs from *S. aureus* 6571 (squares), 2760 (triangles) and 2900 (diamonds) or controls *H. pylori* OMVs (hexagons), rapamycin (circles) or non‐stimulated cells (grey circles). Data show three biological replicates with average ± SEM. Triplicate images were captured per treatment for each biological replicate, with >50 cells per biological replicate counted. **P < 0.01, ***P < 0.001, ****P < 0.0001 compared to NS control unless indicated with a bracket (One‐way ANOVA with Tukey's multiple comparisons test).

To investigate the ability of *S. aureus* MVs to induce autophagy, we transiently transfected A549 lung epithelial cells with a GFP‐expressing microtubule‐associated protein light chain 3 (LC3‐GFP) construct, which is a central protein in the autophagy pathway. Fluorescently labelled MVs from *S. aureus* strains 6571, 2760 and 2900 or the positive controls rapamycin or *H. pylori* OMVs were added to LC3‐GFP expressing cells for 1, 2, 3 or 4 h and the formation of LC3‐GFP autophagic puncta were examined by confocal microscopy (Figure 6). MVs were observed in A549 cells from 1 h post stimulation (Supplementary Figure 4(a)). A time‐dependent increase in LC3‐GFP puncta formation was observed in MV‐stimulated A549 cells (Supplementary Figure 4(a); Figure 6(a)) that reached significant levels 4 h post stimulation (Supplementary Figure 4(b); Figure 6(b)) indicating that MVs induce autophagosome formation in host cells. To assess whether MVs could be degraded via autophagy, A549 cells were pre‐treated with bafilomycin A1 (BafA1) prior to incubation with MVs to inhibit autophagosome degradation and therefore the degradation of MVs (Figure 6(a)). Confocal microscopy showed the accumulation of LC3‐GFP puncta in MV‐stimulated cells pre‐treated with BafA1 (Figure 6(a)), which was quantified and was found to be statistically significant (Figure 6(b)), indicating that MVs activate and are degraded by autophagy. Furthermore, MVs were seen to colocalize with LC3‐GFP puncta in BafA1‐treated cells, indicating that they were present within autophagosomes whose degradation was inhibited.

**FIGURE 7:**
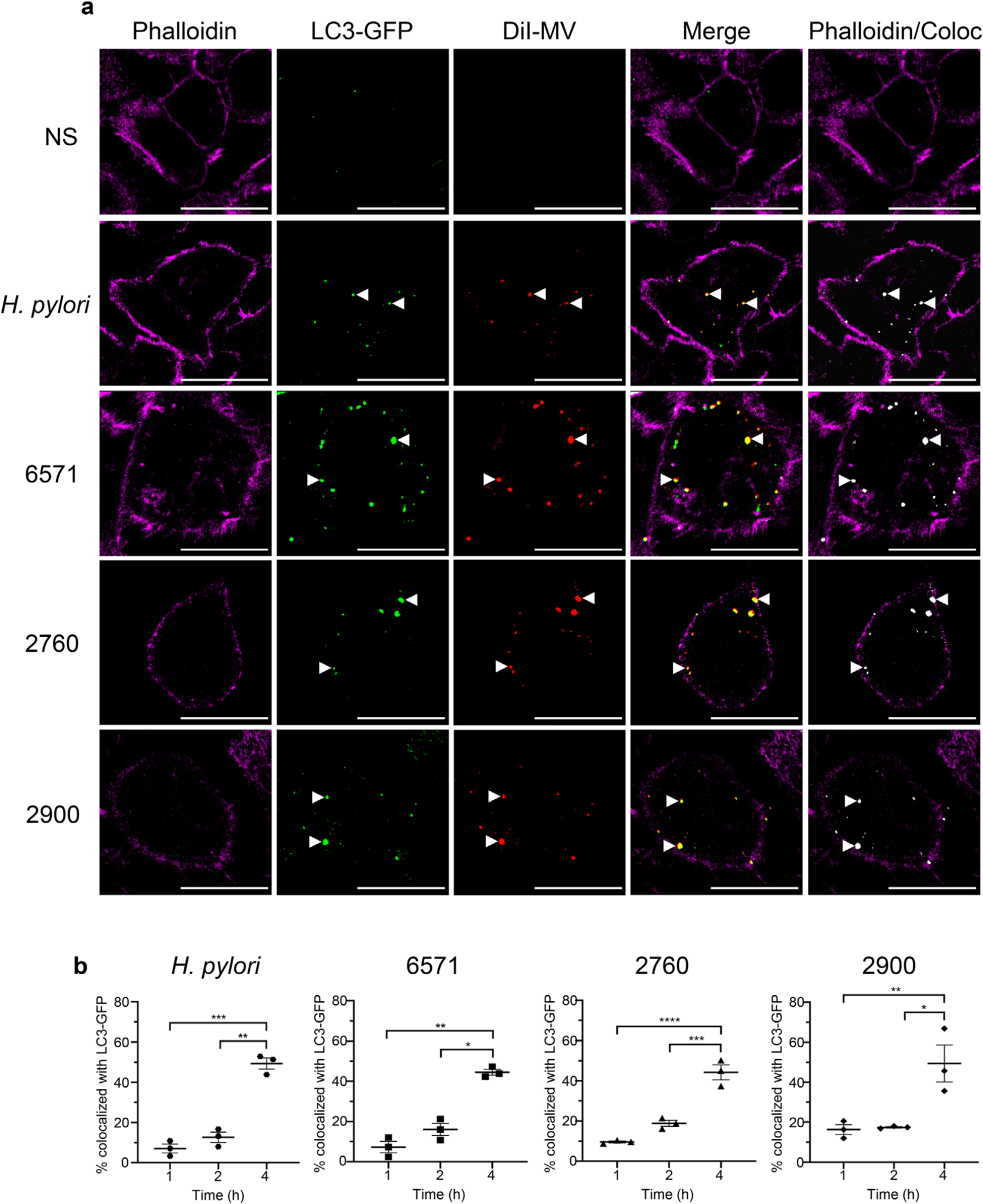
MVs colocalize with LC3‐GFP puncta. (a) Single confocal plane of A549 cells stained with the cytoskeletal marker phalloidin Alexa Fluor 680 (magenta), showing intracellular LC3‐GFP puncta (green) and DiI‐labelled 6571, 2760 or 2900 MVs (red). Merged images show colocalization of LC3‐GFP puncta and MVs (yellow) within the cell cytoskeleton (magenta). Colocalization of LC3‐GFP with MVs was analyzed using Imaris (Bitplane) and detection of colocalized regions (coloc) is shown in white. Arrow heads indicate colocalized LC3‐GFP puncta with DiI‐labelled MVs or OMVs. Controls include *H. pylori* OMVs (red) or non‐stimulated cells. Images are representative of three biological replicates. Scale bar = 20 μm. (b) Percentage of colocalization between LC3‐GFP puncta and *S. aureus* MVs or *H. pylori* OMVs at 1 h, 2 h and 4 h stimulation. Data show three biological replicates with average ± SEM. Triplicate images were captured per treatment for each biological replicate, with >50 cells per biological replicate counted. *P < 0.05, **P < 0.01, ***P < 0.001, ****P < 0.0001 (One‐way ANOVA with Tukey's multiple comparisons test).

We next confirmed the intracellular location of MVs with LC3‐GFP autophagosomes (Figure 7). A549 cells expressing LC3‐GFP were stimulated with DiI‐labelled MVs and a single confocal plane within the cell was analyzed (Figure 7). Phalloidin staining of the cell perimeter of A549 cells was used to confirm the intracellular location of MVs and LC3‐GFP puncta, while colocalization analysis using Imaris (Bitplane) revealed that MVs and LC3‐GFP colocalized within the cell (Figure 7(a)). Quantification of the percentage of MVs that colocalized with LC3‐GFP puncta demonstrated a significant increase in colocalization in a time‐dependent manner, which was particularly evident at 4 h post stimulation with MVs (Figure 7(b)). Taken together, these findings demonstrate for the first time the ability of MVs produced by a Gram‐positive bacterial species to induce the cellular degradation pathway of autophagy once within host cells, which results in facilitating their clearance from the host.

## 4 DISCUSSION

The biogenesis, contents and immunostimulatory properties of OMVs produced by Gram‐negative bacteria have been extensively studied over the past fifty years (Kaparakis‐Liaskos & Ferrero, 2015; Zavan et al., 2020). In contrast, these aspects of MVs, which were identified as being produced by Gram‐positive bacteria only ten years ago, remain poorly understood. In this study, we characterized the production, composition and innate immune receptor activation of MVs isolated from three *S. aureus* strains; a MRSA clinical isolate, a MSSA clinical isolate and a lab‐adapted strain. We identified that MVs produced by all three *S. aureus* strains contained protein, DNA, RNA and peptidoglycan, and thereby confirming that Gram‐positive bacteria package peptidoglycan into MVs. We quantified the amount of protein, DNA, RNA and peptidoglycan associated with a defined number of MVs from each strain, revealing strain‐dependent variation in the quantity of protein and RNA carried by MVs (Figure 3). Furthermore, we demonstrated that MAMPs delivered to non‐phagocytic host cells by MVs activated innate immune receptors TLR2, 7, 8, 9 and NOD2, ultimately resulting in the production of NF‐κB and pro‐inflammatory cytokine release, suggesting that *S. aureus* MVs contribute to host cell inflammation. Finally, we found that once *S. aureus* MVs enter lung epithelial cells, they induce the formation of LC3‐GPF puncta and are associated with autophagosomes, demonstrating for the first time the ability of MVs produced by a Gram‐positive bacterial species to induce the cellular degradation pathway of autophagy. This suggests a novel mechanism for Gram‐positive bacterial MVs in the activation of autophagy to facilitate their degradation in host cells.

We examined MVs from all strains by TEM and quantified their production by NanoSight and ZetaView NTA (Figure 1, Supplementary Figure 2). Interestingly, the lab‐adapted 6571 strain produced significantly more MVs than the clinical bacteraemia isolates (Figure 1). Reduced MV production by *S. aureus* strains has been linked to poorer patient outcomes in clinical bacteraemia cases (Dey et al., 2020). Several genes have been reported to regulate OMV production in Gram‐negative bacteria (Baarda et al., 2019; Nevermann et al., 2019; Roier et al., 2016); conversely few studies have identified genes that regulate MV production by Gram‐positive bacteria (Resch et al., 2016). The *S. aureus* 6571 lab adapted strain, having gone through multiple passages, may have acquired genetic mutations that affect the regulation of MV production. Indeed, a mutation in the *sfp* gene of a *B. subtilis* laboratory strain has been linked to overproduction of MVs compared to wild type strains (Brown et al., 2014). In *S. aureus*, phenol‐soluble modulins have been implicated in the regulation of MV production and deletion of the *psmα* gene reduced vesiculation (Wang et al., 2018). It is therefore possible that genetic differences between the strains in this study affect regulatory factors that control MV production.

An alternative mechanism of MV biogenesis in Gram‐positive bacteria was recently proposed that occurs via prophage‐encoded endolysin that leads to cell lysis (Toyofuku et al., 2017). Prophage‐mediated cell lysis has also been identified as a mechanism of MV biogenesis in *S. aureus* (Andreoni et al., 2019). It is therefore possible that the 6571 strain may harbour a prophage that leads to increased MV production. However, since reduced viability is not observed in the 6571 strain compared to the other strains (Figure 1), it is unlikely that prophage‐mediated cell lysis is the cause of hypervesiculation.

Inter‐strain differences in MV morphology were also observed, whereby elliptical MVs produced by strain 2900 were identified by TEM (Figure 1). Consistent with our findings, elliptical OMVs from *Acinetobacter baylii* (Fulsundar et al., 2014) and irregular shaped OMVs from *Neisseria meningitidis* (Bjerre et al., 2000) have been reported. The latter study identified that vesicle morphology correlated with the amount of lipopolysaccharide (LPS) in the OMV membrane (Bjerre et al., 2000). Therefore, it could be inferred that variation in MV morphology may be due to membrane‐spanning components such as lipoproteins or lipoteichoic acids. Examination of the differences in membrane composition among strains and how this influences MV morphology will be the focus of future research.

Although it was previously thought that MVs from Gram‐positive bacteria did not carry nucleic acids (Dorward & Garon, 1990), recent studies have demonstrated that MVs are associated with DNA (Andreoni et al., 2019; Liao et al., 2014) and RNA (Choi et al., 2018; Resch et al., 2016). Our characterization reveals that *S. aureus* MVs do carry both DNA and RNA (Figures 2 and 3), and DNA was detected on the vesicle surface and within the MV lumen, where it is protected from DNase degradation (Figure 2). This is consistent with studies demonstrating that OMVs contain DNA that is protected from degradation within the vesicle lumen (Bitto et al., 2017; Pérez‐Cruz et al., 2013). OMVs from pathogenic Gram‐negative bacteria have been shown to facilitate intra‐ and inter‐species transfer of antibiotic resistance and virulence genes (Fulsundar et al., 2014; Rumbo et al., 2011; Yaron et al., 2000), therefore there may be the potential for the MV‐mediated transfer of genetic material between Gram‐positive bacteria, however this remains to be elucidated by future studies.

Our data identifies the presence of small RNA associated with *S. aureus* MVs (Figure 2). Small RNAs associated with eukaryotic EVs have been extensively reported and shown to have functional effects on the transcriptional regulation of target cells (Sun et al., 2016; Tominaga et al., 2015). In comparison, little is known about RNAs in prokaryotic vesicles. There have been few studies identifying sRNAs associated with OMVs (Blenkiron et al., 2016; Choi et al., 2017; Ghosal et al., 2015; Koeppen et al., 2016; Malabirade et al., 2018) and limited reports of RNA detected with Gram‐positive bacterial MVs (Choi et al., 2018; Resch et al., 2016; Rodriguez & Kuehn 2020), including sRNA species (Choi et al., 2018). Our findings add further evidence to the presence of sRNA associated with Gram‐positive bacterial MVs. The biological functions of sRNA carried by OMVs and MVs remain unclear, however recent studies have suggested a role for OMV‐bound sRNA in inter‐bacterial communication (Ho et al., 2015) and host‐pathogen interactions (Choi et al., 2017; Koeppen et al., 2016). Small RNAs have been isolated from *S. aureus* bacteria that are involved in regulating the bacterial response to antimicrobial exposure (Howden et al., 2013), suggesting MV‐bound RNAs may serve a similar function. Further investigations are required to characterize MV‐associated sRNA to understand their biological functions.

We identified that *S. aureus* MVs contain immunostimulatory cargo, including identifying the presence of peptidoglycan carried by MVs. The ability of MVs to deliver immunostimulatory DNA, RNA and peptidoglycan into epithelial cells to activate the intracellular PRRs for these ligands has not been previously shown. Here we demonstrate that MVs are therefore detected by the innate immune receptors TLR2, 7, 8, 9 and NOD2, which recognize the MAMPs lipoprotein and lipoteichoic acid (TLR2), RNA (TLR7 and 8), DNA (TLR9) and peptidoglycan (NOD2) (Girardin et al., 2003; Jurk et al., 2002; Latz et al., 2004; Müller‐Anstett et al., 2010; Takeuchi et al., 1999). Thus, our data highlight the immunostimulatory effects of the cargo carried by *S. aureus* MVs and identifies the ability of MVs to deliver nucleic acids and peptidoglycan into epithelial cells to activate intracellular innate immune receptors and drive an innate immune response. We also demonstrate the ability of *S. aureus* MVs to induce cytokine and chemokine responses in human lung epithelial cells, including the neutrophil chemotactic factor IL‐8, the leukocyte‐recruiting chemokine CCL2, and the pro‐inflammatory cytokine IL‐6 (Figure 5). Consistent with our findings, recent studies have reported *S. aureus* MVs induce a range of pro‐inflammatory cytokines *in vitro* and *in vivo* (Tartaglia et al., 2018) and mediate cytotoxicity of epithelial cells (Thay et al., 2013). Further evidence of the innate immune‐activating properties of *S. aureus* MVs has been demonstrated in recent studies, revealing their ability to activate the NLRP3 (NOD‐, LRR‐ and pyrin domain‐containing protein 3) inflammasome (Wang et al., 2020) and TLR‐mediated interferon‐β responses in macrophages (Rodriguez & Kuehn 2020). Together these studies support our findings that *S. aureus* MVs play an inflammatory role in host‐pathogen interactions mediated by their immunostimulatory cargo that can activate innate immune receptors to induce pro‐inflammatory cytokine responses.

Autophagy is a cellular degradation pathway that can be activated by the intracellular innate immune receptors NOD1 or NOD2 in response to bacterial peptidoglycan (Travassos et al., 2010). We have previously demonstrated that OMVs from *H. pylori* and *P. aeruginosa* function as a novel mechanism to deliver peptidoglycan to NOD1, inducing autophagy and mediating NOD1‐driven inflammatory responses in host cells (Irving et al., 2014). Subsequently, NOD1‐dependent activation of autophagy by OMVs from other Gram‐negative species has also been shown (Elluri et al., 2014; Losier et al., 2019). However, the activation of autophagy by Gram‐positive bacterial MVs has not yet been reported. Having determined that *S. aureus* MVs activate NOD2, we sought to investigate the ability of *S. aureus* MVs to induce an autophagic response. Our data reveals that MVs are internalized by human lung epithelial cells and colocalize with LC3‐expressing autophagosomes, a key component of the autophagy pathway, resulting in a significant increase in autophagosome formation over time (Figure 6 and 7). Furthermore, we showed that inhibiting phagolysosomal degradation using bafilomycin A1 leads to the accumulation of *S. aureus* MVs within LC3‐labelled autophagosomes (Figure 6), suggesting that once intracellular, MVs enter the autophagosomal pathway to facilitate their cellular degradation. Our findings therefore reveal a novel mechanism whereby intracellular MVs are degraded by the host, via the cellular degradation pathway of autophagy. This also provides insight into how MV antigens may be degraded to ultimately facilitate the presentation of foreign antigens and generate a MV‐specific immune response.

**FIGURE 8:**
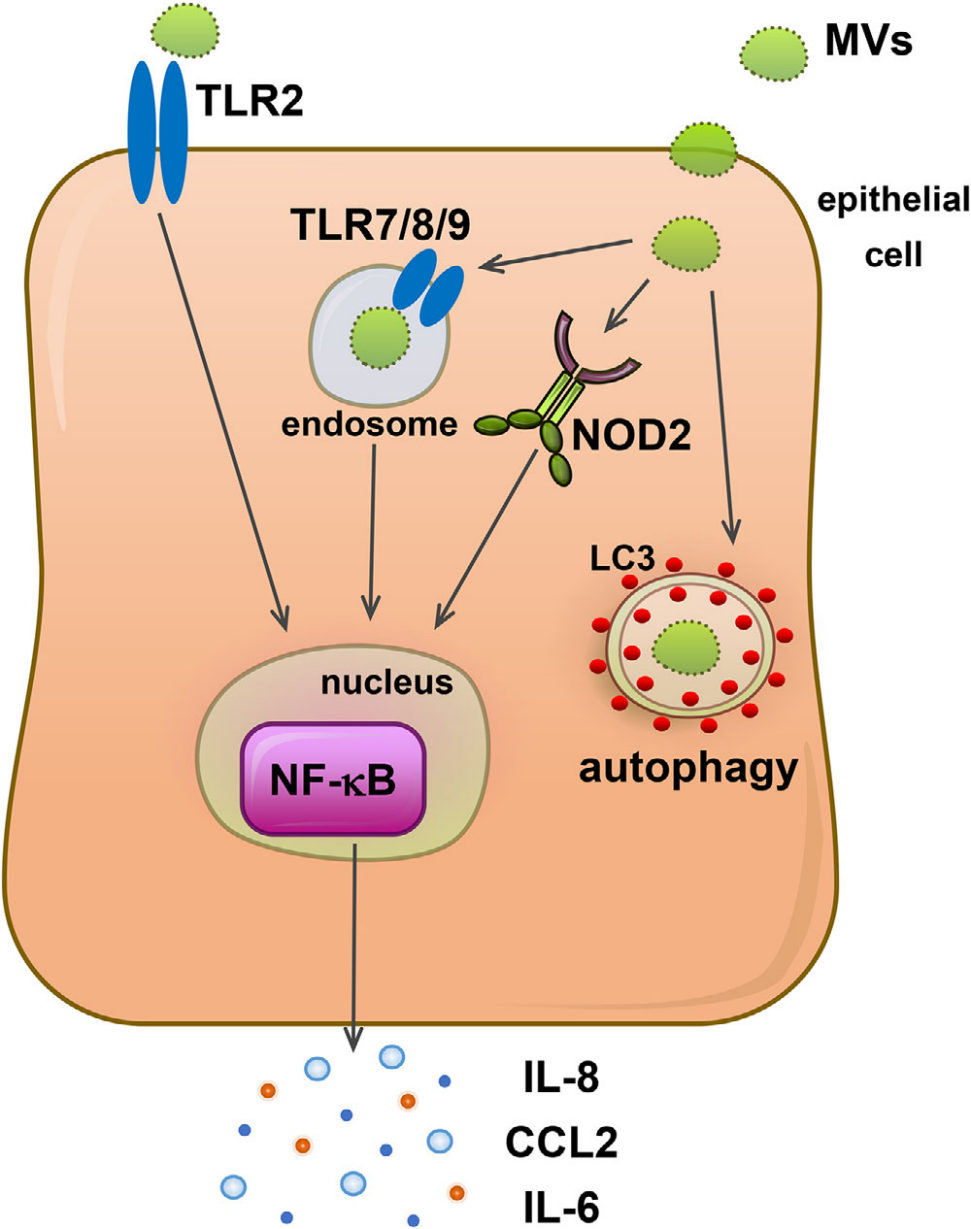
Model of immune detection, inflammatory signaling and intracellular fate of MVs produced by Gram‐positive bacteria. MVs produced by Gram‐positive bacteria can interact with PRRs expressed on the surface of epithelial cells. Interaction of *S. aureus* MVs with epithelial cells results in the activation of TLR2 located at the host cell surface. Furthermore, upon entry into host epithelial cells, MV‐associated RNA, DNA and peptidoglycan cargo is detected by the endosomal PRRs TLR7, 8 and 9, and the cytoplasmic receptor NOD2, respectively. Activation of surface and cytoplasmic host PRRs by MV‐associated cargo results in the activation of NF‐κB and the production and release of pro‐inflammatory cytokines and chemokines to facilitate pathogenesis in the host. Furthermore, intracellular detection of MVs leads to their sequestration into autophagosomes and their subsequent degradation via the host cellular degradation pathway of autophagy to enable clearance of MVs from host cells.

Our findings identify the mechanisms whereby MVs produced by Gram‐positive bacteria activate PRRs to induce an inflammatory response in host epithelial cells and their subsequent intracellular fate (Figure 8). Upon interaction with host epithelial cells, *S. aureus* MVs are detected by the membrane bound PRR TLR2. MV entry into host cells facilitates their interaction with the endosomal receptors for nucleic acids, TLR7, 8 and 9, and the cytoplasmic receptor for peptidoglycan, NOD2, leading to the production of pro‐inflammatory cytokines and chemokines (Figure 8). The intracellular detection of MVs triggers the formation of autophagosomes and the processing of MVs via the host cellular degradation pathway of autophagy, to ultimately facilitate their clearance from the host (Figure 8). Collectively, these findings reveal the ability of *S. aureus* MVs to deliver immunostimulatory DNA, RNA and peptidoglycan cargo into host epithelial cells to activate PRRs, cytokine and chemokine responses and their intracellular degradation via autophagy. These findings advance our understanding of the immunostimulatory functions and intracellular fate of the cargo delivered to host epithelial cells by MVs, and provide insights into mechanisms by which other Gram‐positive bacteria may use MVs to mediate pathogenesis and cause disease in the host.

## DISCLOSURE OF INTEREST

The authors report no conflict of interest.

## Supporting information

Supplementary Figure 1 TEM of fractions from OptiPrep ultracentrifugal density gradient for the purification of *S. aureus* MVs. MVs were observed in fractions 2–12 (white arrows). Fractions 2–9 were pooled to obtain purified MVs, while fractions 10–12 were discarded to eliminate contaminating cellular debris and media components (black arrows). Scale bar = 0.5 μm.

Supplementary Figure 2 Size distribution of *S. aureus* MVs isolated from **(a)** 6571, **(b)** 2760 and **(c)** 2900 determined by ZetaView. Data shows the mean of three pooled biological replicates.

Supplementary Figure 3 **(a)** Equivalent amounts of MVs (1 × 10^10^) from *S. aureus* 6571, 2760 and 2900 were examined using Western immunoblot using an anti‐*S. aureus* antibody to detect MV protein. Bacterial whole‐cell lysates (WC; 10 μg protein) served as positive controls. Bands present predominantly in 2760 MVs but not in MVs from other strains are highlighted with a black arrow, while bands present in 6571 and 2760 MVs but not in 2900 MVs are indicated with a black diamond. M = Precision Plus Protein standard (Bio‐Rad Laboratories). **(b)** Equivalent amounts of MVs (1 × 10^10^) from *S. aureus* 6571, 2760 and 2900 were examined using Western immunoblot using an anti‐peptidoglycan antibody to detect MV‐associated peptidoglycan. Bands present in 6571 and 2900 but not 2760 MVs are highlighted with an arrow, while bands present in 6571 and 2760 but not 2900 MVs are highlighted with a diamond. Peptidoglycan derived from *S. aureus* (*Sa*PG; 40 μg; Sigma Aldrich, USA) served as a positive control.

Supplementary Figure 4 Formation of autophagosomes is time‐dependent. (a) LC3‐GFP (green)‐expressing A549 cells were stimulated with DiI‐labelled 6571, 2760 or 2900 MVs or *H. pylori* OMVs (red; positive control) for 1, 2 or 3 h. Intracellular MVs and OMVs were observed 1, 2 and 3 h post stimulation. Early stages of LC3‐GFP puncta formation at 2 and 3 h is evidenced by colocalization between LC3‐GFP puncta and MVs/OMVs (yellow; indicated by arrow heads). Non‐stimulated cells (NS) served as a negative control. Cell nuclei were stained using DAPI (blue). Images are representative of 3 biological replicates. Scale bar = 20 μm. (b) Quantification of LC3‐GFP puncta formation at 1, 2 and 3 h incubation in non‐stimulated cells (grey circles), cells treated with *H. pylori* OMVs (black hexagons), or MVs from *S. aureus* 6571 (black squares), 2760 (black triangles) and 2900 (black diamonds). Data shows three biological replicates with average ± SEM. Triplicate images were captured per treatment for each biological replicate, with >50 cells per biological replicate counted.
