# Supplementary figures and images for: Staphylococcus aureus membrane vesicles contain immunostimulatory DNA, RNA and peptidoglycan that activate innate immune receptors and induce autophagy
Source: J Extracell Vesicles. 2021 Apr 1;10(6):e12080. doi: 10.1002/jev2.12080 (PMC8015888; doi:10.1002/jev2.12080)

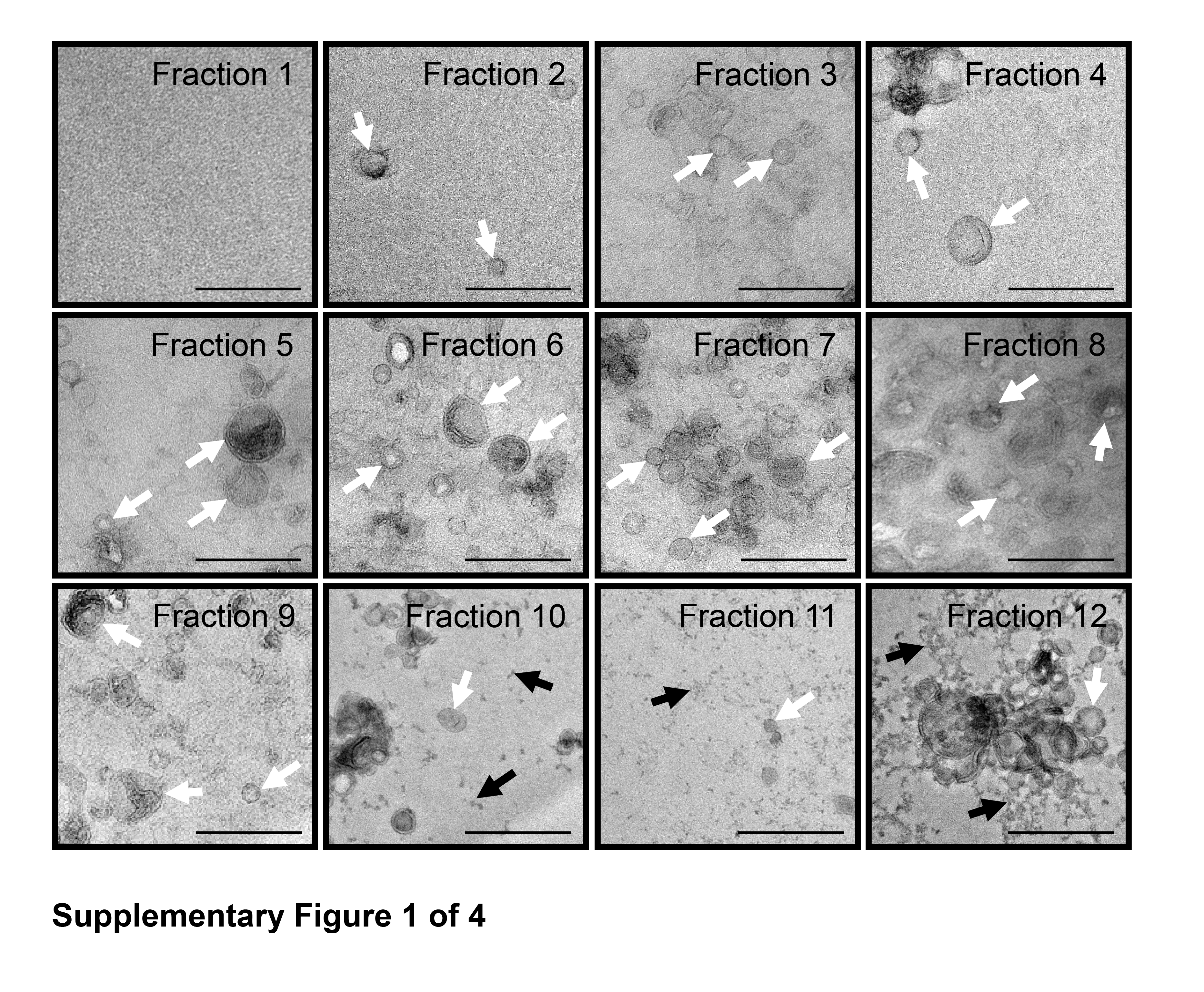

Supplement: Supplementary file 1 — Supplementary Figure 1 TEM of fractions from OptiPrep ultracentrifugal density gradient for the purification of S. aureus MVs. MVs were observed in fractions 2–12 (white arrows). Fractions 2–9 were pooled to obtain purified MVs, while fractions 10–12 were discarded to eliminate contaminating cellular debris and media components (black arrows). Scale bar = 0.5 μm. [file JEV2-10-e12080-s002.tif]

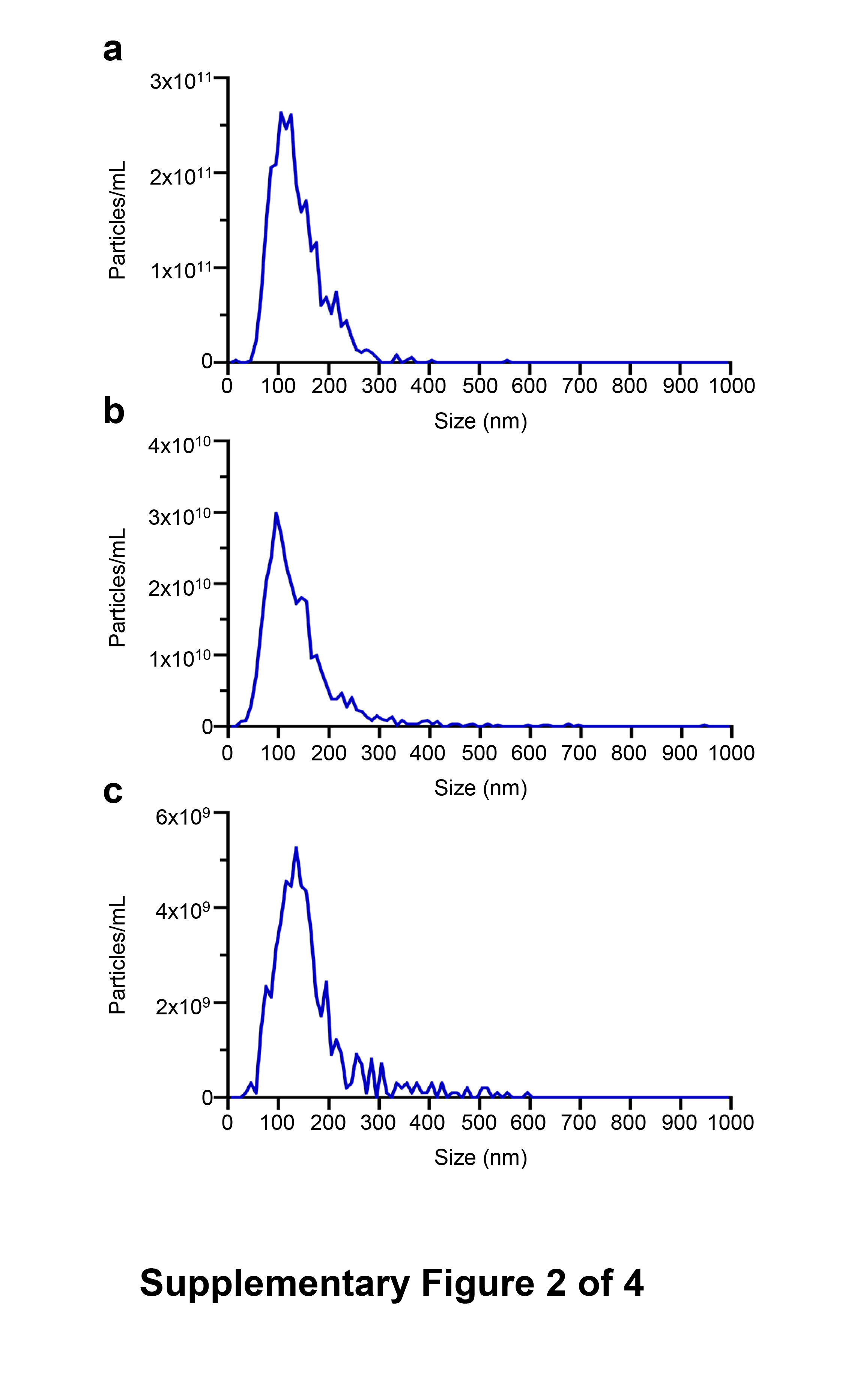

Supplement: Supplementary file 2 — Supplementary Figure 2 Size distribution of S. aureus MVs isolated from (a) 6571, (b) 2760 and (c) 2900 determined by ZetaView. Data shows the mean of three pooled biological replicates. [file JEV2-10-e12080-s001.tif]

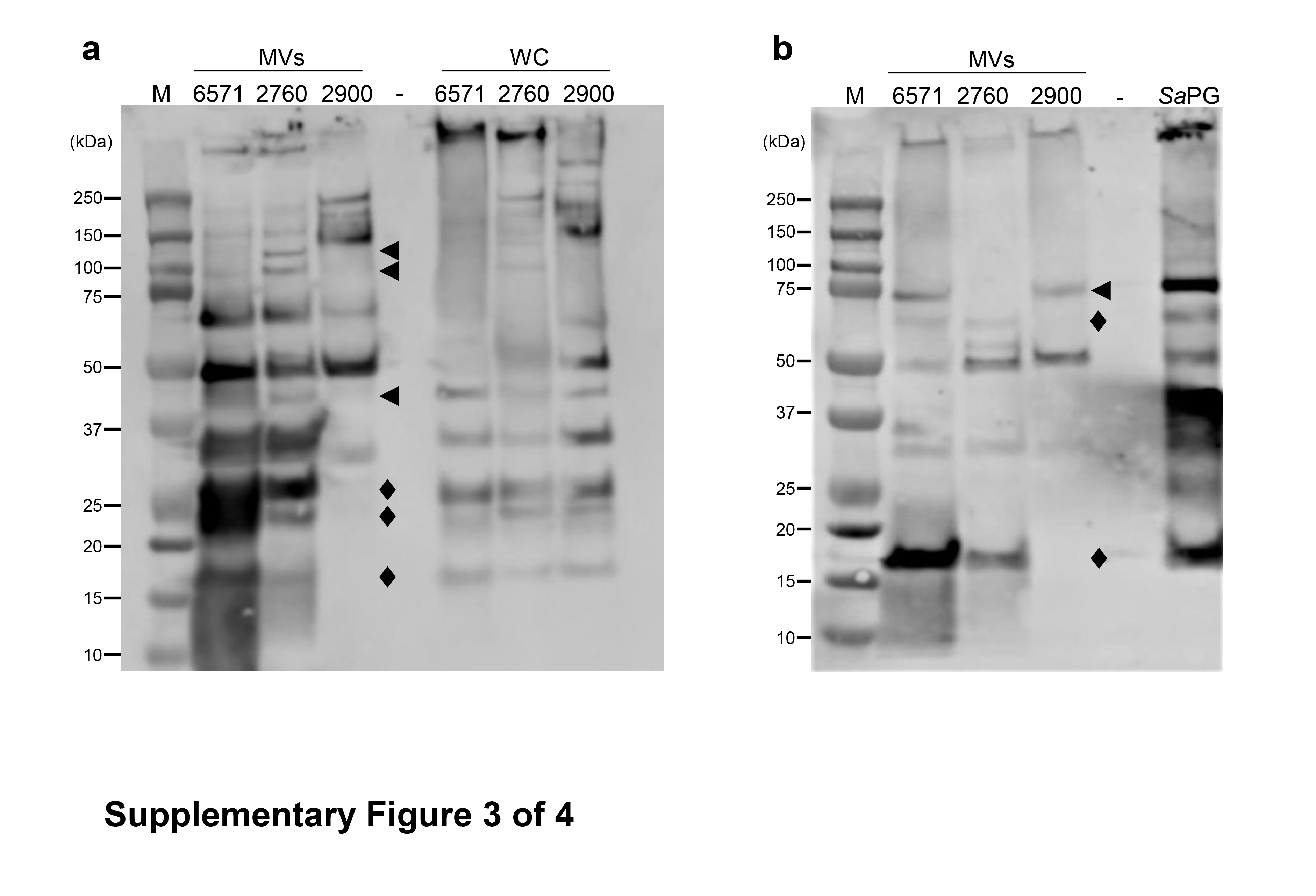

Supplement: Supplementary file 3 — Supplementary Figure 3 (a) Equivalent amounts of MVs (1 × 10^10^) from S. aureus 6571, 2760 and 2900 were examined using Western immunoblot using an anti‐S. aureus antibody to detect MV protein. Bacterial whole‐cell lysates (WC; 10 μg protein) served as positive controls. Bands present predominantly in 2760 MVs but not in MVs from other strains are highlighted with a black arrow, while bands present in 6571 and 2760 MVs but not in 2900 MVs are indicated with a black diamond. M = Precision Plus Protein standard (Bio‐Rad Laboratories). (b) Equivalent amounts of MVs (1 × 10^10^) from S. aureus 6571, 2760 and 2900 were examined using Western immunoblot using an anti‐peptidoglycan antibody to detect MV‐associated peptidoglycan. Bands present in 6571 and 2900 but not 2760 MVs are highlighted with an arrow, while bands present in 6571 and 2760 but not 2900 MVs are highlighted with a diamond. Peptidoglycan derived from S. aureus (SaPG; 40 μg; Sigma Aldrich, USA) served as a positive control. [file JEV2-10-e12080-s004.tif]

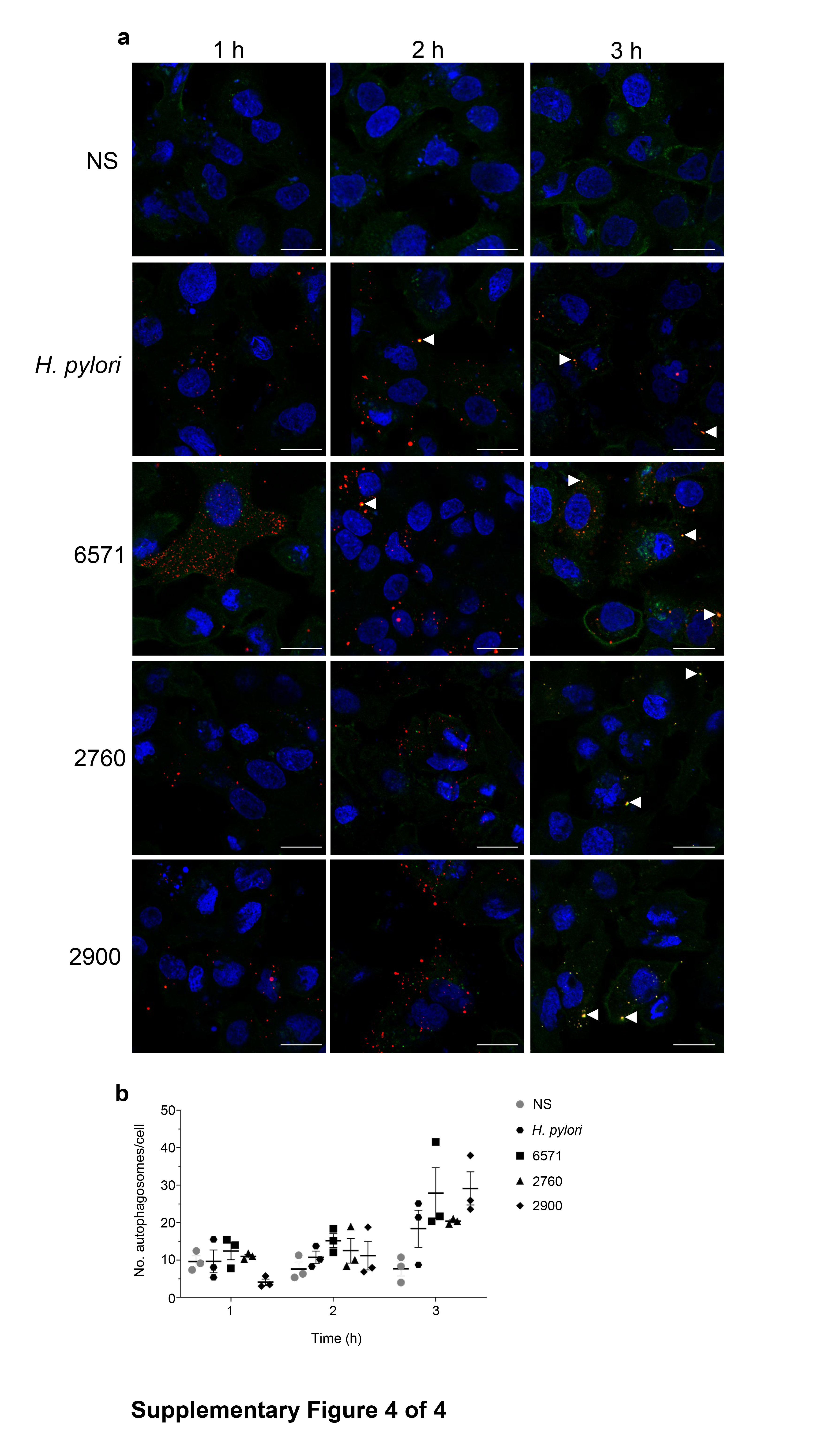

Supplement: Supplementary file 4 — Supplementary Figure 4 Formation of autophagosomes is time‐dependent. (a) LC3‐GFP (green)‐expressing A549 cells were stimulated with DiI‐labelled 6571, 2760 or 2900 MVs or H. pylori OMVs (red; positive control) for 1, 2 or 3 h. Intracellular MVs and OMVs were observed 1, 2 and 3 h post stimulation. Early stages of LC3‐GFP puncta formation at 2 and 3 h is evidenced by colocalization between LC3‐GFP puncta and MVs/OMVs (yellow; indicated by arrow heads). Non‐stimulated cells (NS) served as a negative control. Cell nuclei were stained using DAPI (blue). Images are representative of 3 biological replicates. Scale bar = 20 μm. (b) Quantification of LC3‐GFP puncta formation at 1, 2 and 3 h incubation in non‐stimulated cells (grey circles), cells treated with H. pylori OMVs (black hexagons), or MVs from S. aureus 6571 (black squares), 2760 (black triangles) and 2900 (black diamonds). Data shows three biological replicates with average ± SEM. Triplicate images were captured per treatment for each biological replicate, with >50 cells per biological replicate counted. [file JEV2-10-e12080-s003.tif]
